# TRPM8-Rap1A Interaction Sites as Critical Determinants for Adhesion and Migration of Prostate and Other Epithelial Cancer Cells

**DOI:** 10.3390/cancers14092261

**Published:** 2022-04-30

**Authors:** Giorgia Chinigò, Guillaume P. Grolez, Madelaine Audero, Alexandre Bokhobza, Michela Bernardini, Julien Cicero, Robert-Alain Toillon, Quentin Bailleul, Luca Visentin, Federico Alessandro Ruffinatti, Guillaume Brysbaert, Marc F. Lensink, Jerome De Ruyck, Anna Rita Cantelmo, Alessandra Fiorio Pla, Dimitra Gkika

**Affiliations:** 1 Department of Life Sciences and Systems Biology, University of Torino, 10123 Torino, Italy; giorgia.chinigo@unito.it (G.C.); madelaine.audero@gmail.com (M.A.); michela.bernardini3@gmail.com (M.B.); luca.visentin@unito.it (L.V.); federicoalessandro.ruffinatti@unito.it (F.A.R.); alessandra.fiorio@unito.it (A.F.P.); 2 INSERM, U1003—PHYCEL—Physiologie Cellulaire, University of Lille, F-59000 Lille, France; grolez.guillaume@gmail.com (G.P.G.); alexandre.bokhobza@inserm.fr (A.B.); quentin.bailleul@univ-lille.fr (Q.B.); anna-rita.cantelmo@univ-lille.fr (A.R.C.); 3 CNRS, INSERM, CHU Lille, Centre Oscar Lambret, UMR 9020-UMR 1277-Canther-Cancer Heterogeneity, Plasticity and Resistance to Therapies, University of Lille, F-59000 Lille, France; julien.cicero@univ-lille.fr (J.C.); robert-alain.toillon@univ-lille.fr (R.-A.T.); 4 UR 2465—Laboratoire de la Barrière Hémato-Encéphalique (LBHE), University of Artois, F-62300 Lens, France; 5 CNRS UMR 8576-UGSF-Unité de Glycobiologie Structurale et Fonctionnelle, University of Lille, 59000 Lille, France; guillaume.brysbaert@univ-lille.fr (G.B.); marc.lensink@univ-lille.fr (M.F.L.); jerome.de-ruyck@univ-lille.fr (J.D.R.); 6 Department of Molecular and Cell Biology, University of California, Berkeley, CA 94720, USA; 7 Institut Universitaire de France (IUF), 75231 Paris, France

**Keywords:** prostate cancer, metastasis, TRPM8, Rap1A, migration, adhesion, GTPase, calcium channel

## Abstract

**Simple Summary:**

Prostate cancer is the second leading cause of cancer death among men. Its poor prognosis is mainly due to metastases, and therefore, it is crucial to understand the mechanisms by which cancer cells can spread throughout the body. In this context, among others, we demonstrated the TRPM8 channel to be a major player due to its ability to impair prostate cancer cell motility. The present work elucidates the molecular mechanism through which TRPM8 exerts its inhibitory effect on cell migration. We found that this effect is mediated by a direct interaction between residues E207 and Y240 in the N-terminal TRPM8 tail and residue Y32 in the Switch I region of the Rap1-GDP bound inactive form. Since these results have also been validated in breast and cervical cancer cells, the TRPM8-Rap1 interaction could constitute a therapeutic target against metastatic progression in several types of tumors.

**Abstract:**

Emerging evidence indicates that the TRPM8 channel plays an important role in prostate cancer (PCa) progression, by impairing the motility of these cancer cells. Here, we reveal a novel facet of PCa motility control via direct protein-protein interaction (PPI) of the channel with the small GTPase Rap1A. The functional interaction of the two proteins was assessed by active Rap1 pull-down assays and live-cell imaging experiments. Molecular modeling analysis allowed the identification of four putative residues involved in TRPM8-Rap1A interaction. Point mutations of these sites impaired PPI as shown by GST-pull-down, co-immunoprecipitation, and PLA experiments and revealed their key functional role in the adhesion and migration of PC3 prostate cancer cells. More precisely, TRPM8 inhibits cell migration and adhesion by trapping Rap1A in its GDP-bound inactive form, thus preventing its activation at the plasma membrane. In particular, residues E207 and Y240 in the sequence of TRPM8 and Y32 in that of Rap1A are critical for the interaction between the two proteins not only in PC3 cells but also in cervical (HeLa) and breast (MCF-7) cancer cells. This study deepens our knowledge of the mechanism through which TRPM8 would exert a protective role in cancer progression and provides new insights into the possible use of TRPM8 as a new therapeutic target in cancer treatment.

## 1. Introduction

Prostate cancer (PCa) is the most frequently diagnosed cancer among men and the second leading cause of cancer death after lung cancer [1]. PCa poor prognosis is mainly due to metastases. Hence, there is an urgent need to deepen our knowledge of the mechanisms through which the primary tumor can acquire more aggressive phenotypes, and therefore, the ability to exit the primary site and establish secondary growth in distant organs through lymphatic or blood circulation. Metastasis fundamentally involves cell migration, a complex and multistep process that subtends coordination between cytoskeleton remodeling, cell-substrate adhesion/detachment, and cellular protrusion/contraction [2,3].

Over the past two decades, an increasing set of data has revealed a pivotal role of ion channels, including TRP channels, in many processes underlying the metastatic cascade, making them promising candidates as both molecular biomarkers and therapeutic targets in cancer therapy [4,5,6,7,8,9]. More specifically, marked changes in TRP protein expression have been associated with the development and progression of several cancers depending on their stage [6,10,11]. Interestingly, although most of the best-characterized roles played by TRP channels in the metastatic cascade rely on their channel activity [12], there is growing evidence of pathways that underlie the interaction of TRP channels with different partner proteins, extending interest in TRPs beyond the field of ion channels [13,14,15,16,17,18,19,20]. In this context, particular attention has been paid to the close bidirectional interplay revealed by TRP channels and small GTPase in all phases of the metastatic cascade, including migration, invasion, and tumor vascularization [21]. In most cases, this functional interplay is mediated by calcium (Ca^2+^) signals, triggered by TRP-mediated Ca^2+^ influx within the cell, which may affect small GTPases activity and their impact on the regulation of cell migration and/or invasion [22,23,24,25,26,27,28,29,30,31,32]. In turn, small GTPase may regulate TRP channels’ intracellular trafficking to the plasma membrane (PM) or act directly on channel gating, thus impacting their biological functions [22,23,31,33,34,35]. However, some data revealed alternative regulatory pathways independent of cation homeostasis, through which TRP-small GTPase interplay may affect cancer progression [33,35,36,37]. 

Among TRP channels, TRPM8 is one of the most intriguing TRP channels involved in PCa progression, due to its specific and characteristic expression profile. Indeed, *TRPM8* expression was shown to increase in both benign prostate hyperplasia (BPH) and in prostate carcinoma cells characterized by high androgen levels [10] but decrease with tumor progression to the late androgen-insensitive invasive stage [38,39]. This specific expression pattern is attributable to an androgen-dependent regulation of *TRPM8* [40,41]. More specifically, *TRPM8* turned out to be a primary androgen response gene [41,42], since the testosterone androgen-receptor complex (AR) is able to directly promote *TRPM8* transcription by binding with androgen-responsive elements present on its gene sequence. Besides the androgen-dependent transcription, testosterone exerts an additional non-genomic rapid effect on TRPM8 channel activity through the AR and translocation of the two proteins in lipid rafts [40]. More specifically, it has been shown that at low concentrations, testosterone can promote the physical interaction between TRPM8 and AR within lipid rafts on the PM and consequently an increased AR-mediated inhibition of TRPM8 activity [40]. Consequently, the anti-androgen therapy by downregulating the expression of AR not only greatly reduces the expression of TRPM8, but also affects the inhibitory action on the control of channel activity, leading PCa cells to an androgen-independent stage in which they relapse with a more aggressive phenotype [39,40]. Consistent with these data, we previously revealed a protective role played by TRPM8 in prostate cancer progression, thanks to its ability to inhibit PCa cell migration in vitro and in vivo [40,43,44,45]. Although some data suggest a role of the Ca^2+^-dependent inactivation of the focal-adhesion kinases (FAK) [46], the exact molecular mechanism by which TRPM8 impairs the motility of epithelial prostate cancer cells is still unclear. 

Interestingly, in a previous study, we unveiled that the anti-metastatic function of TRPM8 may be also extended to endothelial cells (ECs), suggesting a TRPM8 involvement also in the angiogenic process [47]. Indeed, endoplasmic reticulum (ER) TRPM8 expression in ECs inhibits cell migration via a direct binding with Rap1A with subsequent inhibition of inside-out β_1_-integrin signaling [47]. 

Here, we questioned whether the same molecular mechanism showed in ECs underlies the TRPM8-mediated inhibition of cell migration observed in epithelial prostate cancer cells. Previous evidence on TRPM8 interactors confirmed the presence of Rap1A among potential TRPM8 partner proteins in mouse prostate [44]. In this work, we demonstrated that in PCa cells TRPM8 exerts its anti-migratory function through a pore-independent pathway involving the binding to the inactive form of Rap1A and the subsequent inhibition of its activity in promoting cell adhesion. Furthermore, we deepened the TRPM8-Rap1A interaction, identifying and functionally validating the residues involved in its mediation on both fronts. Notably, this interesting new facet of TRPM8 as a Rap1 inhibitor is not limited to PCa but can be extended to other epithelial cancer cell lines. The complete characterization of the molecular mechanisms by which TRPM8 performs its anti-metastatic function could provide new and important insights into view, of the possible and successful use of TRPM8 as a pharmaceutical target in the treatment of advanced stages of metastatic cancer.

## 2. Materials and Methods

### 2.1. Chemicals and Drugs

Icilin used as a specific TRPM8 agonist was purchased from Tocris (Bio-Techne, Noyal Châtillon sur Seiche, France), dissolved in DMSO to a final concentration of 20 mM, and stored at −20 °C. Fura2-AM calcium probe used in Ca^2+^ imaging experiments was purchased from Invitrogen Ltd. (Fisher Scientific UK Ltd., Loughborough, UK), dissolved in DMSO to a final concentration of 1 mM, and stored at −20 °C. Thapsigargin was purchased from Sigma-Aldrich (Sigma-Aldrich, Milan, Italy), dissolved in DMSO to a final concentration of 2 mM, and stored at −20 °C. 

### 2.2. Molecular Modeling

As the N- and C-tail of the TRPM8 structure was not totally solved by cryo-EM [48], we modeled this tetrameric structure by homology modeling. For this, BLASTP was used to obtain templates similar to the TRPM8 sequence. One template was identified with a similarity of over 30% and corresponds to the cryo-EM structure of TRPA1 (Resolution: 4.2 Å, PDB ID: 3J9P) [49]. Using the sequence of TRPM8 and the identified templates, we performed the modeling of one monomer and then the full tetrameric form using Modeler [50]. The global structure was subsequently minimized to remove steric clashes.

This model was further used to predict the interaction between TRPM8 and Rap1A, for which a crystal structure existed (Resolution: 1.9 Å, PDB ID: 1C1Y) [51]. This prediction was obtained using a protein-protein docking algorithm and more specifically the ClusPro2.0 [52] and InterEvDock [53] webservers. Seven residues at the interface between TRPM8 and Rap1A were visually selected, looking at the two first models of InterevDock and three among the five first best models of ClusPro 2 (TRPM8: Glu207, His259, Leu262, Tyr240, Val263; Rap1A: Lys31, Tyr32). For each model, a Residue Interaction Network (RIN) was generated with an in-house C program. A RIN is defined as a network in which each node is a residue, and each edge is a contact detected between two residues in the 3D structure. Here, residue-residue contacts were detected when the distance between them was found between 2.5 Å and 5 Å. Residue Centrality Analyses (RCA) were then performed with the RINspector app [54,55] for Cytoscape [56,57] and Z-scores ≥ 3 were considered as central residues. These residues were shown to be essential for the folding or function of a structure [58,59], therefore, constituting good candidates for mutagenesis experiments. Here, we focused on the residues at the interface, central to the quaternary structure and essential for the interaction. Among the visually selected residues listed before, we listed those that were central in at least one of the five models. Four residues out of the seven selected residues were found central in at least one model (TRPM8: Glu207, Tyr240; Rap1A: Lys31, Tyr32) and were chosen for mutagenesis.

### 2.3. Cell Cultures

In this work, we used human prostate adenocarcinoma cell line (PC3, ATCC), derived from bone metastasis of prostate cancer, human cervical cancer cell line (HeLa, ECACC), and human breast adenocarcinoma cell line (MCF-7, ECACC). PC3 stably expressing for TRPM8 (PC3M8) were generated by stable transfection of pcDNA4-TRPM8 vector as described in [40]. Cell lines were grown in monolayers using FalconTM plates in RPMI 1640 (Invitrogen, Fisher Scientific UK Ltd., Loughborough, UK), MEM (Euroclone, Milan, Italy) and DMEM High Glucose (Euroclone, Milan, Italy) growth medium supplemented with 10% of fetal bovine serum (Pan Biotech, Aidenbach, Germany), L-glutamine (5 mM; Sigma-Aldrich, Saint-Quentin Fallavier, France) and PenStrep (100 mg/mL; Sigma-Aldrich, France) for PC3, HeLa, and MCF-7 cells, respectively. PC3-TRPM8 cells were submitted to Zeocin (InvivoGen, San Diego, CA, USA) selection (100 μg/mL) every two passages. Cells were cultured in a humidified atmosphere consisting of 95% air and 5% CO_2_ at 37 °C and they were used at passages 2 to 15.

### 2.4. Molecular Biology

The constructs used for this work were His-tagged-hTRPM8pcDNA4 [60], pGEX6p2, pGEX6p2-TRPM8Nt, pGEX6p2-TRPM8Ct [45], peGFP-Rap1A, pMT2SM-HA-Rap1A, pCMN-TNT-Rap1A (for all Rap constructs wild-type (wt) and S17N) [48], pEGFP-RBD_RalGDS_ [61]. 

Simple and double mutagenesis for TRPM8 (E207A and Y240A) and Rap1A (K31A and Y32A) are summarized in Table 1 and were conducted by site-directed mutagenesis using in vitro mutagenesis (QuikChange Site-directed Mutagenesis kit, Agilent Technologies-Stratagene products and Q5^®^ Site-Directed Mutagenesis Kit).

Gene overexpression in PC3 cells was obtained using Lipofectamine^TM^ 3000 reagents (Invitrogen, Fisher Scientific UK Ltd., Loughborough, UK), according to manufacturer’s instructions, in RPMI 1640 (Invitrogen, Fisher Scientific UK Ltd., Loughborough, UK) 10% FBS growth medium. GFP tagged or human influenza agglutinin (HA) tagged-Rap1 (wild-type, Y32A, K31A, S17N, S17N Y32A and S17N K31A; 0.625–5 µg for transfection) and GFP-tagged as control (5 µg for transfection); pEGFP-RBD_RalGDS_ (1 μg for transfection). Cells were tested 24 h or 48 h after transfection, depending on the assay (24 h for Ca^2+^ imaging experiments, migration and adhesion assays, active Rap1 pull-down assays and live-cells imaging experiments; 48 h for GST-pull-down assays and immunoprecipitation assays).

### 2.5. Western Blot Analysis

Cells were plated in 6-well culture plates, grown to a confluency of 80%, and then silenced and/or overexpressed according to the protocol described below. Before cell lysis, multiwell plates were kept on ice, washed twice in ice-cold PBS, and then cells were lysed in the presence of RIPA buffer (Pierce^®^ RIPA Buffer, Thermo Fisher Scientific, Waltham, MA, USA); 1% Triton X-100, 1% Na deoxycholate, 150 mM NaCl, 10 mM NaKPO_4_, pH 7.2) and anti-protease inhibitor cocktail (Sigma-Aldrich P2714, Milan, Italy; 1:10). Lysates were vortexed, kept for 10 min on ice, sonicated, and then centrifuged at 4 °C for 13 min at 13,000× *g*. Protein concentrations of the supernatant were determined using a bicinchoninic acid (BCA) kit (Sigma-Aldrich, Milan, Italy) following the manufacturer’s instructions. 

Thirty micrograms of lysates were resuspended in SDS loading buffer, heated 5 min at 95 °C or 30 min at 37°, and then loaded and separated on 4–20% pre-cast SDS-PAGE gels (Thermo Fisher Scientific, Waltham, MA, USA) at 130 mV-150 mV. Quick transfer on Polyvinylidene fluoride membranes (PVDF) membranes at 3A, 2.5 V for 15 min was followed. Subsequently, membranes were blocked 30 min in TBST (20 mM Tris, 150 mM NaCl, 0.1% Tween 20, pH 7.6) 5% BSA and then incubated in agitation overnight at 4 °C with rabbit anti–Rap1 (Thermo Fisher scientific 1,862,344; 1:1000 in TBST 5% BSA 0.02% sodium azide), rabbit anti-TRPM8 (Ab 109,308; 1:800 in TBST 1% BSA+ sodium azide), and mouse anti-β-actin (Sigma-Aldrich A5316; 1:1000 in TBST 1% BSA 0.02% sodium azide) antibodies. Membranes were then washed with TBST and incubated with the appropriate horseradish peroxidase (HPR)–conjugated antibodies (anti-mouse HRP Thermo Fisher Scientific #31430, Waltham, MA, USA; 1:10,000 in TBST 1% BSA and anti-rabbit HRP Santa Cruz Biotechnology #2054, Santa Cruz, CA, USA; 1:15,000 in TBST 5% BSA). Chemiluminescence assays were conducted using the Western Lightning Plus-Enhanced Chemiluminescence Substrate (PerkinElmer, Milan, Italy). To quantify differences in protein expression, the ratio between overexpressed samples and PC3 wt (each normalized on actin) was evaluated, using Fiji (ImageJ software).

### 2.6. GST-Fusion Protein and Pull-Down Assay

TRPM8 N- and C-terminal peptides fused with the GST tag were produced from plasmids pGEX6p-2-TRPM8-Nt and pGEX6p-2-TRPM8-Ct and then purified as described previously [44]. GST-fused purified proteins were then incubated with HEK or PC3 cell lysates transfected with pEGFP-Rap1 S17N, pEGFP-Rap1 S17N K31, pEGFP-Rap1 S17N Y32 plasmids.

For the direct interaction assay, Rap1 wt and mutants’ coding sequence were in vitro translated using the TNT Quick Coupled Transcription/Translation Systems Kit (Promega, Madison, WI, USA). Briefly, the coding sequences of the proteins of interest were inserted in the pCMV-TNT vectors (Promega, Madison, WI, USA) as EcoR1-Not1 fragments to produce and translate them in vitro, according to the manufacturer’s instructions. 

Subsequently, TRPM8 GST-fusion proteins were incubated overnight at 4 °C with cell lysates or in vitro translated proteins. Beads were then washed 4× with the IP buffer and bound proteins were eluted in SDS-PAGE loading buffer, heated at 70 °C for 10 min, and separated on 4–20% pre-cast SDS-PAGE gels (Bio-Rad Laboratories Inc., Marnes-la-Coquette, France) starting from 0 mV to 130 mV. Quick transfer on PVDF membranes at 2.5 V for 20 min was followed. Membranes were then blocked 2 h in TNT (0.1 M Tris-Cl, 150 mM NaCl, 0.1% Tween 20, pH 7.5) 5% BSA and analyzed by immunoblotting using rabbit anti-Rap1 (Thermo Fisher Scientific #MA5-15052, Waltham, MA, USA; 1:500 in 1.5% BSA) or mouse anti-GFP (Clontech #632,380, Takara Bio Company, Saint-Germain-en-Laye, France; 1:1000 in 1% milk) antibody for Rap1 detection. Membranes were incubated with primary antibodies overnight at 4 °C in agitation, washed three times with TNT 1× (15 mM Tris, 140 mM NaCl, 0.05% Tween, pH = 7.4) and incubated 1 h with anti-rabbit secondary antibody (Santa Cruz Biotechnology, Santa Cruz, CA, USA; 1:20,000 in 2% BSA) or anti-mouse secondary antibody (Santa Cruz Biotechnology, Santa Cruz, CA, USA; 1:50,000 in 1% milk). Chemiluminescence assays were conducted using the SuperSignal West Dura chemiluminescent substrate (Thermo Fischer Scientific, Waltham, MA, USA). TRPM8-Rap1 interaction was quantified as the ratio between IP_Rap1_ and IP_M8_ (both normalized on their own total lysate) and then it was normalized on TRPM8-Rap1 N17 interaction, used as control. Each experiment was repeated at least three times.

### 2.7. Co-Immunoprecipitation Assay

PC3M8 were seeded in 100 mm dishes (30 × 10^4^ cells/dish) and, 48 h after plating, they were transfected with 5 μg of GFP-Rap1S17N, GFP-Rap1S17N_K31A, GFP-Rap1S17N_Y32A, and GFP- pcDNA3 plasmids. 48 h after transfection, cells were lysed and incubated for 20–30 min on ice in lysis buffer (30 mM Tris HCl, 150 mM NaCl, 1% CHAPS, pH = 7.5, and anti-protease cocktail; Sigma-Aldrich). After centrifugation (12,000× *g* for 10 min at 4 °C) of the lysates, protein concentration was determined by BCA assay kit (Thermo Fisher Scientific, Waltham, MA, USA) and at least 800 μg of proteins in 500 μL of IP buffer (150 mM NaCl, 20 mM NaH_2_PO_4_, pH 8) were incubated overnight in rotation at 4 °C with 50 μL of His-Tag Dynabeads^®^ (Thermo Fisher Scientific, Waltham, MA, USA), previously extensively washed in 500 μL IP buffer. 

Samples were then washed four times in IP buffer, eluted in SDS loading buffer, heated at 95 °C for 5 min or at 37° for 30 min, and then separated on 4–20% or 7.5% pre-cast SDS-PAGE gels (Bio-Rad Laboratories Inc., Marnes-la-Coquette, France) starting from 90 mV to 130 mV. Total lysate for positive control was prepared with 50 μg of proteins suspended in an SDS loading buffer. Quick transfer on PVDF membranes at 3 A, 2.5 V for 15 min was followed. Membranes were then blocked for 1 h in TNT 5% milk and analyzed by immunoblotting using mouse anti-GFP (Clontech, #632,380, Takara Bio Company, Saint-Germain-en-Laye, France; 1:1000 in 1% milk) antibody for Rap1′s detection, and rabbit anti-myc (Abcam #9106, Cambridge, UK; 1:1000 in 1% milk) or rabbit anti-TRPM8 (Abcam #109,308, Cambridge, UK; 1:800 in TBST 1% BSA+ sodium azide) antibodies for TRPM8′s detection. Membranes were incubated with primary antibodies overnight at 4 °C in agitation, washed three times with TNT 1X, and incubated for 1 h with secondary antibodies (1:50,000 in 1% milk). Chemiluminescence assays were conducted using the SuperSignal West Dura chemiluminescent substrate (Thermo Fischer Scientific, Waltham, MA, USA). Each experiment was repeated at least three times.

### 2.8. Proximity Ligation Assay (PLA)

Proximity Ligation Assays (PLA) were performed using Duolink (R) In Situ Red-Starter Kit Goat/Rabbit (Sigma-Aldrich, Saint-Quentin Fallavier, France). PC3 cells were seeded (10 × 10^4^ cells/dish) on confocal FluoroDish (World Precision Instruments) and then transfected or not with TRPM8 wt or E207A Y240A and Rap1 N17 (1 μg for each plasmid). After fixation, cells were permeabilized with 4% platelet-activating factor for 10 min and then incubated in the blocking buffer (PBS 4% BSA) for 1 h and 30 min at room temperature. Primary antibodies diluted in the antibody diluents (rabbit anti–TRPM8 antibody, N571644, 1:200; Antibodies-online; mouse anti-GFP 1:200; Takara) were then added to the cells overnight at 4 °C in a humidified chamber. The rest of the protocol was performed according to the manufacturer’s instructions. Recordings were performed by confocal imaging (LSM880; ZEISS) using z-stack superposition (Zen black 2010 software; ZEISS). Appropriate controls were performed by incubating PC3 cells with both primary antibodies separately. In order to assess where the interaction between the two proteins occurs, ER staining was assessed 24 h time after transfecting the cells with 0.3 µg of Ds-Red2 plasmid fused to the ER targeting sequence of calreticulin (Ds-Red2 ER probe, Takara Bio Europe SAS, Saint-Germain-en-Laye, France) in bottom glass dishes.

### 2.9. Live-Cell Imaging Experiments

To study the endogenous activity of Rap1, we performed a set of live-cell GTPase activity assays using the GFP-RBD_RalGDS_ probe. The first set of live-cell imaging experiments was performed on PC3 and PC3M8 and a second one on PC3 overexpressing M8 wt or M8 E207A Y240A (1 µg plasmid for transfection). 

Cells were seeded on bottom glass dishes coated with 1% gelatin (5 × 10^4^ cells/dish) and 48–72 h after plating were transfected (or co-transfected) with 0.5 μg of pEGFP-RalGDS-RBD using Lipofectamine^TM^ 3000 reagents (Invitrogen, Fisher Scientific UK Ltd., Loughborough, UK) in RPMI 1640 0% FBS growth medium. 24 h after transfection, cells were imaged using an LSM 780 confocal microscope equipped with an argon laser. Live cells were kept at 37 °C and 5% CO_2_ for all experiments, and confocal images were acquired every minute using the “perfect focus” option to maintain the same focal plane. After 10 min of acquisition, cells were stimulated with 10 µM icilin. 

Images were acquired using ZEN software and analyzed offline with ImageJ (National Institutes of Health, Bethesda, MA, USA). As a direct indication of Rap1 activation, we measured the relative cytoplasmic translocation of GFP-RBD_RalGDS_, as previously described [61]. Briefly, for each cell, we checked at least four ROIs of identical size around an area of distinct PM fluorescence and around an adjoining region of cytosol without membrane encroachment [47]. The fluorescence intensity (I) was determined for these ROIs at each time point. Relative cytoplasmic translocation (R) was calculated as R = (I_cs_ − I_m_)/I_m_, where I_m_ and I_cs_ refer to the fluorescence intensities of the regions of interest in the membrane and in the cytosolic compartment, respectively. About 15–20 cells from at least three independent experiments were analyzed for each experimental condition. Statistical analysis of the data was performed using a Kruskal–Wallis test with a Dunn’s *post-hoc* test.

### 2.10. Active Rap1 Pull-Down and Detection Assay

To study Rap1 activation we used the Active Rap1 Pull-Down and Detection kit from Thermo Fisher Scientific, according to the manufacturer’s instructions as previously described [47]. The detection of GTP-bound Rap1 GTPase was obtained through specific protein interactions with the RalGDS protein-binding domain. 

PC3 and PC3 overexpressing TRPM8 cells were seeded in 100 mm dishes and transfected (or co-transfected in ratio 1:1) with Rap1 mutant plasmids or TRPM8 double mutant (see conditions in Results). Pulled-downs were performed 24 h after transfection, treating or not the cells with 10 μM icilin for 15 min. Pull-down samples and total lysates used as control were separated on 4–20% pre-cast SDS-PAGE gels (Thermo Fisher Scientific, Waltham, MA, USA) at 130 mV. Quick transfer on PVDF membranes at 3 A, 2.5 V for 15 min was followed. Membranes were then blocked for 30 min in TBST 5% BSA and analyzed by immunoblotting using rabbit anti-Rap1 (Thermo Fisher Scientific #MA5-15,052, Waltham, MA, USA; 1:1000 in TBST 5% BSA 0.02% sodium azide) antibody for Rap1′s detection. Membranes were incubated with primary antibodies overnight at 4 °C in agitation, washed three times with TBST 1X, and incubated 1 h with secondary antibody (anti-rabbit HRP Santa Cruz Biotechnology #2054, Santa Cruz, CA, USA; 1:15,000 in TBST 5% BSA). Chemiluminescence assays were conducted using the Western Lightning Plus-ECL (PerkinElmer, Milan, Italy).

Rap1 activation was quantified as the ratio between the GTP-bound Rap1 fraction (pulled-down) and the amount of Rap1 in total lysates, next normalized on PC3 or PC3M8 as control. At least three independent experiments for each condition were performed and statistical significance was assessed by a one sample paired *t*-test or RM one-way ANOVA without Geisser–Greenhouse correction and with Dunnett’s *post-hoc* test. 

### 2.11. Cytosolic and ER Ca^2+^ Imaging

Cells were seeded on glass coverslips at a density of 0.5 × 10^4^ cells/dish and were transfected with overexpression plasmids (his tagged-hTRPM8pcDNA4 wt, Y905A, and E207A Y240A; GFP-tagged hTRPM8pcDNA4 wt, empty pcDNA4) at least 48 h after seeding. 

Cytosolic Ca^2+^ signals were monitored by loading the cells (24 h after transfection) at 37 °C for 30 min with 2 μM Fura-2 AM (Invitrogen), a ratiometric probe used for cytosolic calcium concentration ([Ca^2+^]_i_) measurements as previously described [62]. During experiments, cells were maintained in a standard extracellular solution of the following composition: 154 mM NaCl, 4 mM KCl, 2 mM CaCl_2_, 1 mM MgCl_2_, 5 mM HEPES, and 5.5 mM glucose (NaOH to adjust pH to 7.4). Fluorescence measurements were made using a Polychrome V spectrofluorimeter (TILL Photonics GmbH, BioRegio, Munich, Germany) attached to an Olympus X51 microscope (Olympus, Tokyo, Japan) and Metafluor Imaging System (Molecular Devices, Sunnyvale, CA, USA) for image acquisition using 3-s intervals. Each fluorescent trace (340/380 nm ratio) represents the mean ± SEM of at least 20 traces obtained from one representative experiment. For each condition, at least three independent experiments were performed. The IgorPro software (Wavematrix, Lake Oswego, OR, USA) was used to further analyze fluorescence traces and to calculate the peak amplitude (fluorescence intensity ratio, measured at 510 nm), determined by the difference between the maximum and minimum values of fluorescence intensity recorded in about 300 s upon agonist administration. We used the agonist-induced slope change as a criterion to distinguish a cell response to an agonist from the background noise. In PC3 overexpressing TRPM8, only responsive cells were considered for the analysis. The Kruskal–Wallis test with a Dunn’s *post-hoc* test was used for the statistical evaluation of the data.

ER Ca^2+^ signals were monitored by transfecting the cells with the GEM-Cepia1er plasmid (1 μg), a Ca^2+^ biosensor targeted to the ER. pCIS GEM-Cepia1er was a gift from Masamitsu Iino (Addgene plasmid #58217; http://n2t.net/addgene:58217 accessed on 25 September 2015; RRID:Addgene_58217) [63]. 24 h after GEM-Cepia1er transfection, images were acquired at a magnification of 40× (Nikon Plan 40X/0.10 objective) using a Nikon Eclipse Ti (Nikon Corporation, Tokyo, Japan) inverted microscope equipped on a system integrated by Crisel Instruments (Rome, Italy), controlled with MetaMorph software (Molecular Devices, Sunnyvale, CA, USA), a CCD camera (Q imaging), a filter wheel (Suter Instrument), and a fluorescence lamp with the following excitation and emission settings: excitation 405 ± 20 nm; emission at 460 ± 50 nm and 525 ± 50 nm. During experiments, cells were maintained in a standard extracellular solution of the following composition: 154 mM NaCl, 4 mM KCl, 2 mM CaCl_2_, 1 mM MgCl_2_, 5 mM HEPES, and 5.5 mM glucose (NaOH to adjust pH to 7.4). Metafluor Imaging System (Molecular Devices, Sunnyvale, CA USA) was used to acquire images at 5-s intervals. The 460/525 nm ratio referring to several ROIs drawn within each cell was recorded and then mediated and normalized on the mean fluorescence signal recorded 100 s before the stimulation (ΔF/F_0_). Consequently, a significant decrease in the ΔF/F_0_ ratio revealed an ER Ca^2+^ store depletion. The well-known sarco/endoplasmic reticulum Ca^2+^ ATPase (SERCA) inhibitor thapsigargin (2 μΜ) was used as a control for ER store depletion. The IgorPro software (Wavematrix, Lake Oswego, OR, USA) was used to further analyze fluorescence traces and to calculate the ER-Ca^2+^ release’s slope upon stimulation by icilin and thapsigargin.

### 2.12. Random Migration Assay

Cells were seeded in 24-well culture plates coated with 1% gelatin (3 × 10^4^ cells/well), using RPMI 1640 10% FBS, and incubated at 37 °C and 5% CO_2_ atmosphere for 24 h. Cells were then transfected with 1 μg of his tagged-hTRPM8pcDNA4 (plasmid wild-type, Y905A, and E207A Y240A) and were treated with 10 μM icilin 24 h after transfection. 

The experiment was performed using a Nikon Eclipse Ti (Nikon Corporation, Tokyo, Japan) inverted microscope equipped with an A.S.I. MS-2000 stage, a System integrated by Crisel Instruments (Rome, Italy) for multi-wavelengths and multi-positions widefield time-lapse, CCD camera (Photometrics) and OkoLab incubator, in order to keep cells at 37 °C and 5% CO_2_. MetaMorph software (Molecular Devices, Sunnyvale, CA, USA) was used to acquire images for 6 h every 10 min, using a Nikon Plan 20X/0.10 objective and a CCD camera.

Image stacks were analyzed with ImageJ software (National Institutes of Health, Bethesda, MA, USA), and about 400 cells/condition from three independent experiments were tracked manually using the MtrackJ plugin. Cells that exited the imaged field or doubled during the time-lapse acquisition interval were excluded from the analysis. Migration rate (μm/min) is obtained by measuring the distance covered by cells between two consequent time points after conversion of pixels to micrometers. The Kruskal–Wallis test with a Dunn’s *post-hoc* test was used for the statistical evaluation of the data.

### 2.13. Transwell Migration Assay

Transwell^®^ permeable supports (6.5 mm inserts with an 8 μm pore polycarbonate membrane) were equilibrated for 20 min at 37 °C using DMEM 0% FBS medium. The equilibrated Transwell^®^ inserts were then placed into 24-well culture plates (Sarstedt, Germany) containing DMEM 10% FBS. 24 h after transfection with his tagged-hTRPM8pcDNA4 (plasmid wild-type, and E207A Y240A) cells were seeded (2.5 × 10^4^ cells/insert) in the Transwell^®^ inserts using DMEM 10% FBS and incubated O/N. Transwell^®^ inserts were then washed twice in PBS and the cells that did not migrate through the membrane pores were removed from the upper side of the membrane using a cotton bud. After washing in PBS, cells were fixed in cold ethanol for 20′ at 4 °C and then stained with DAPI in PBS for 15 min at 37 °C. Cells that migrated through the membrane pores were then counted using a Nikon Eclipse Ti (Nikon Corporation, Tokyo, Japan) inverted microscope (4× objective).

### 2.14. Adhesion Assay

Cell adhesion was evaluated on 96-well culture plates using a coating of 1% gelatin. 24 h after transfection (see conditions in Results), cells were detached using trypsin for 3 min, carefully counted, and seeded (0.3 × 10^4^ cells/well) in 100 μL RPMI 10% FBS growth medium in the presence or absence of 10 μM icilin. 

Cells were kept at 37 °C and 5% CO_2_ for 60 min and then washed twice with PBS with Ca^2+^ and Mg^2+^. Cells were fixed in ethanol for 20 min at 4 °C and then washed twice with PBS with Ca^2+^ and Mg^2+^. Staining of cell nuclei was performed using 0.2 mM Hoechst for 15 min at room temperature. Then cells were washed again and kept in PBS. 

Image acquisition was performed using a Nikon Eclipse Ti (Nikon Corporation, Tokyo, Japan) microscope with a 4× objective and cell nuclei were counted using a homemade automatic cell count macro in ImageJ. At least eight wells for each condition were analyzed in each independent experiment and at least three independent experiments were performed for each condition tested. Statistical significance was assessed using the Kruskal–Wallis test with a Dunn’s *post-hoc* test.

### 2.15. Cell Viability Assay

Cells were seeded in 6-well plates (Sarstedt, Germany) and transfected with 0.625 μg of his tagged-hTRPM8pcDNA4 (TRPM8 wild-type, and E207A Y240A) using Lipofectamine^TM^ 3000 reagents (Invitrogen, Fisher Scientific UK Ltd., Loughborough, UK), according to manufacturer’s instructions; 6 h after transfection, the cells were detached, counted accurately, and plated (0.5 × 10^4^ cells/well) in 96-well black-bottom polystyrene plates (Greiner Bio-One, Austria). 18, 42, and 66 h after incubation at 37 °C (i.e., 24, 48, and 72 h after transfection), cell viability was assessed using CellTiter-Glo^®^ Luminescent Cell Viability Assay (Promega, Madison, WI, USA), following manufacturer’s instructions. Luminescence was recorded using a microplate reader (FilterMax F5, Multi-Mode Microplate Reader, Molecular Devices) and then analyzed as being proportional to the number of viable cells. For each condition, eight replicates were set up and three independent experiments were performed.

### 2.16. Confocal Analysis

Cells (10 × 10^4^ cells/dish) were seeded in 40 mm diameter bottom glass dishes and were transfected with GFP-tagged hTRPM8pcDNA4 wt (1 μg) 24 h after seeding. Cells were incubated at 37 °C for 24 h and then washed and incubated with ER-Tracker^TM^ Red (500 nM, Molecular probes^®^, Invitrogen, Fisher Scientific UK Ltd., Loughborough, UK) for 30 min. After incubation, cells were washed twice with Hanks’ Balanced Salt Solution (HBSS from Euroclone, Milan, Italy) in order to wash off the excess probe and fixed in 4% paraformaldehyde (PAF) at 37 °C for 2 min. Images were acquired through a Leica TCS SP8 confocal system (Leica Microsystems GmbH, Wetzlar, Germany) equipped with an HCX PL APO 63X/1.4 NA oil-immersion objective. GFP-TRPM8 was excited at 488 nm, and ER-Tracker^TM^ Red at 561 nm with DPSS lasers. Images were acquired on the three coordinates of the space (XYZ planes) with a resolution of 0.081 μm × 0.081 μm × 0.299 μm and were processed and analyzed with ImageJ software (National Institutes of Health, Bethesda, MA, USA) in order to assess TRPM8 localization in the ER.

Similarly, PC3 cells overexpressing both TRPM8 and Rap1A N17 were analyzed by PLA (see Section 2.8, above) and co-localization of PLA dots with ER staining was assessed (Ds-Red ER probe).

### 2.17. Immunohistochemistry

Immunostaining was performed on normal and cancerous prostate tissue arrays (Biomax Inc.). Slides were deparaffinized, rehydrated, and heated in citrate buffer pH 6 for antigenic retrieval. After blocking for endogenous peroxidase with PBS-G Tween (0.2 M Glycine + tween 20 0.1%), slides were incubated with goat anti-TRPM8 (ABIN572229, dilution: 1:50) and rabbit anti-Rap1A (ABIN2854404, dilution: 1:50). Following three washing steps, slides were incubated with the corresponding secondary antibodies: Rhodamine Red-X -labeled anti-goat (Jackson ImmunoResearch, dilution: 1:50) and Alexa Fluor 488-labeled anti-rabbit (Jackson ImmunoResearch, dilution: 1:500). Nuclei were counterstained with DAPI (Invitrogen, Life Technologies, Ghent, Belgium). Confocal imaging was performed using an LSM 880 confocal microscope (Carl Zeiss AiryScan, Munich, Germany). 

### 2.18. Bioinformatic Analysis

#### 2.18.1. Detection of Mutations

We retrieved TCGA MuTect2 mutational data from the UCSC Xena databases [64] Using an ad-hoc pipeline [65] we also retrieved up-to-date clinical metadata regarding all patients included in the TCGA study. By combining the metadata with the mutation dataset, we were able to run filtering to find any mutations of interest. For normalization purposes, the protein lengths of Rap1A and TRPM8 were set to be 184 and 1104 amino acids, respectively [66,67].

#### 2.18.2. Differential Expression Analysis

We retrieved normalized expression data from the UCSC Xena databases, including samples from TCGA, the TARGET and the GTEx projects. We also retrieved metadata for the GTEx samples from the same database portal and used the same TCGA metadata as in the previous analysis. Samples from the TARGET cohort were removed. The samples were divided into “Breast”, “Prostate” and “Uterus” cohorts:

- GTEx samples labeled as “Breast” and TCGA samples labeled as “TCGA-BRCA” composed the “Breast” dataset;
- GTEx samples labeled as “Prostate” and TCGA samples labeled as “TCGA-PRAD” composed the “Prostate” dataset;
- GTEx samples labeled as “Uterus” or “Cervix Uteri” and TCGA samples labeled as “TCGA-UCEC” composed the “Uterus” dataset.
- The samples were labeled as “healthy”, “metastatic”, or “not metastatic” based on these criteria (“Metastatic” labeling):
- If the sample was from the TCGA cohort and had an entry in the AJCC “m” category (both clinical or pathologic) reflecting the metastatic nature of the sample, it was labeled as “metastatic”;
- If the sample was labeled as having AJCC stage “IV” or equivalent, it was labeled as “metastatic”;
- If the sample was labeled with a FIGO stage of “III” or “IV”, it was labeled as “metastatic”;
- If the sample was from the GTEx cohort, it was labeled as “healthy”;
- All other samples were labeled as “not metastatic”.

Differential expression analysis was carried out separately on the expression of the two genes in the three distinct cancer cohorts. Normality and homoscedasticity requirements were checked for all cohorts. In particular, for each cohort, residuals within each group were assumed to be normally distributed about the group mean if they met either of the following conditions:

- Produced a non-significant *p*-value under the Kolmogorov-Smirnov two-sided test with the null hypothesis of being drawn from a normal distribution;
- Had sample numerosity greater than 30.

If normality assumptions were not met, the differential expression in the cohort was computed with the Kruskal–Wallis Rank Sum Test followed by Dunn’s All-Pairs Rank Comparison *post-hoc* test with Bonferroni multiple comparison correction. Otherwise, an ANOVA (Analysis of Variance) model was fitted and followed by Tukey’s Honest Significant Differences *post-hoc* test. Homoscedasticity of the data was checked for all cohorts analyzed with ANOVA models, and all proved satisfactory.

On the contrary, the Rap1A Breast “Metastatic”, Rap1A Prostate “Metastatic”, TRPM8 Breast “Metastatic” and TRPM8 Prostate “Metastatic” cohorts did not meet the normality assumptions.

All code used to perform the bioinformatics analysis, as well as further information on how to reproduce the analysis is available on GitHub gists at this URL [68].

### 2.19. Statistical Analysis

Data are normalized and expressed as means ± SEM when data are normally distributed and as median ± interquartile range when they do not show a normal distribution (Shapiro-Wilk normality test). Statistical analyses were performed using GraphPad Prism software (Graph Pad Software Inc., San Diego, CA, USA) and differences with a *p*-value < 0.05 were considered statistically significant (*: *p* ≤ 0.05; **: *p* ≤ 0.01; ***: *p* ≤ 0.001; ****: *p* ≤ 0.0001). Statistical significance between different conditions was determined by analysis of variance (1 way or 2 way ANOVA-Kruskal–Wallis test) followed by Tukey’s or Dunn’s multiple comparisons *post-hoc* test to compare more than two conditions to each other in GST pull-down, Ca^2+^ imaging, and live-cell imaging experiments. The Student’s *t*-test or Mann–Whitney test were used to evaluate significance between only two different conditions within one experiment (GST pull-down, immunoprecipitation assay, PLA, active Rap1 pull-down, random migration, and adhesion performed on TRPM8 wt and TRPM8 Y905A/TRPM8 E207A Y240A). One sample *t*-tests or RM one-way ANOVA without Geisser-Greenhouse correction and with Dunnett’s *post-hoc* test were used to evaluate significance in migration, adhesion, and active Rap1 pull-down assays.

## 3. Results

### 3.1. TRPM8 Inhibits PC3 Migration and Adhesion Regardless of Channel Activity

**Figure 1:**
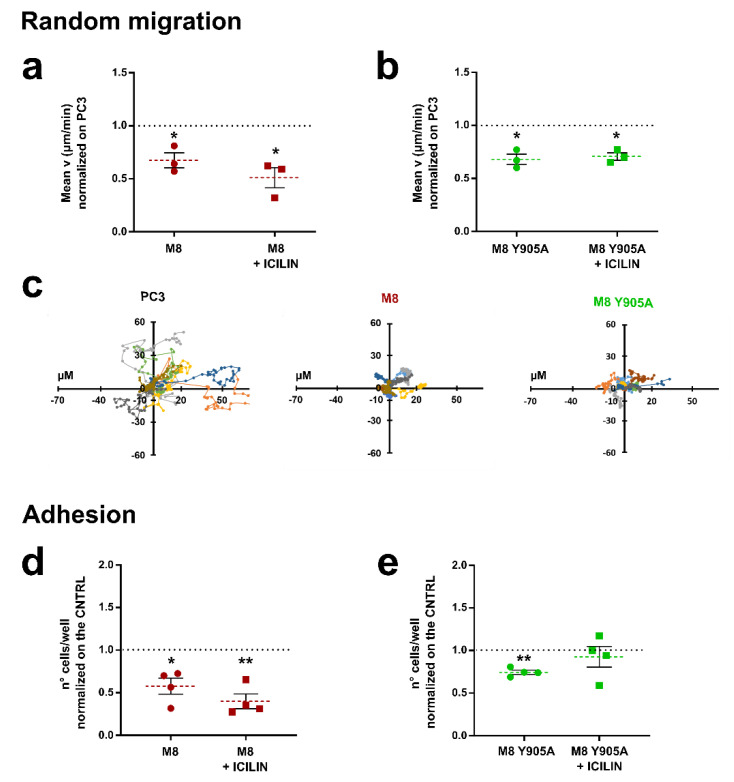
TRPM8 inhibits cell migration and adhesion independently of its channel activity. (**a**) Quantification of random migration assays on PC3 overexpressing TRPM8, treated or not with 10 µM icilin. Data are normalized on PC3 or PC3 treated with icilin (controls represented by dot line) and displayed as mean ± SEM of three independent experiments. Statistical significance versus PC3 or PC3 treated with icilin, *: *p* < 0.05 (One sample *t*-test). (**b**) Quantification of random migration assays on PC3 overexpressing TRPM8 pore mutant, treated or not with 10 µM icilin. Data are normalized on PC3 or PC3 treated with icilin (controls represented by dot line) and displayed as mean ± SEM of three independent experiments. Statistical significance versus PC3 or PC3 treated with icilin,*: *p* < 0.05 (One sample *t*-test). (**c**) Representative migration plots of PC3 (left panel), PC3 overexpressing TRPM8 (central panel) and PC3 overexpressing TRPM8 Y902A (right panel). Each line represents the trajectory of one cell within a 6 h period. Data shown refer to one field of one representative experiment out of three repeats (10 cells for each condition). (**d**) Quantification of adhesion assays on PC3 overexpressing TRPM8 treated or not with icilin (10 μΜ). Data are normalized on PC3 or PC3 treated with icilin (controls represented by dot line) and displayed as mean ± SEM of four independent experiments. Statistical significance versus PC3 or PC3 treated with icilin *: *p* < 0.05; **: *p* < 0.01 (One sample *t*-test). (**e**) Quantification of adhesion assays on PC3 overexpressing TRPM8 pore mutant treated or not with icilin (10 μΜ). Data are normalized on PC3 or PC3 treated with icilin (controls represented by dot line) and displayed as mean ± SEM of four independent experiments. Statistical significance versus PC3 or PC3 treated with icilin **: *p* < 0.01 (One sample *t*-test).

Random migration assays performed on PCa cells from bone metastasis (PC3) confirmed that TRPM8 overexpression leads to a significant reduction in terms of cell migration speed both in the presence and absence of TRPM8 specific agonist icilin (10 μM) (Figure 1a,c). To elucidate the underlying mechanism of this biological effect and, taking into account the inhibition observed even in the absence of channel activation, we first assessed whether the ionic flux generated by TRPM8 activation played a role in cell migration. We performed random migration assays in PC3 overexpressing TRPM8 wild-type (wt) or a TRPM8 inactive pore mutant (Y905A), which completely impairs channel activity (Figure S1). As shown in Figure 1b,c, cell migration was significantly inhibited in PC3 overexpressing TRPM8 Y905A both in the presence and absence of the channel agonist.

To better characterize TRPM8’s inhibitory role, we performed adhesion assays transfecting PC3 cells with TRPM8 wt and plating them on 1% gelatin-coated wells, in the presence or absence of icilin (10 μM). In accordance with cell migration data, overexpression of TRPM8 is correlated with a significant decrease in cell adhesion, even in the absence of the agonist, indicating a role of this channel in inhibiting prostate cancer cell adhesion (Figure 1d). In the absence of the agonist, overexpression of TRPM8 pore mutant affected cell adhesion to the same extent as TRPM8 wt (Figure 1e), confirming that the biological effect of TRPM8 on PC3 cells migration and adhesion are not imputable to the pore function of TRPM8 protein (Figure 1b,e). However, interestingly icilin (10 µΜ) stimulation did not inhibit cell adhesion in PC3 cells overexpressing the TRPM8 pore mutant (Figure 1e), suggesting a Ca^2+^ involvement.

### 3.2. TRPM8 Inhibits Rap1′s Activity by Intracellularly Sequestering GDP-Bound Rap1

**Figure 2:**
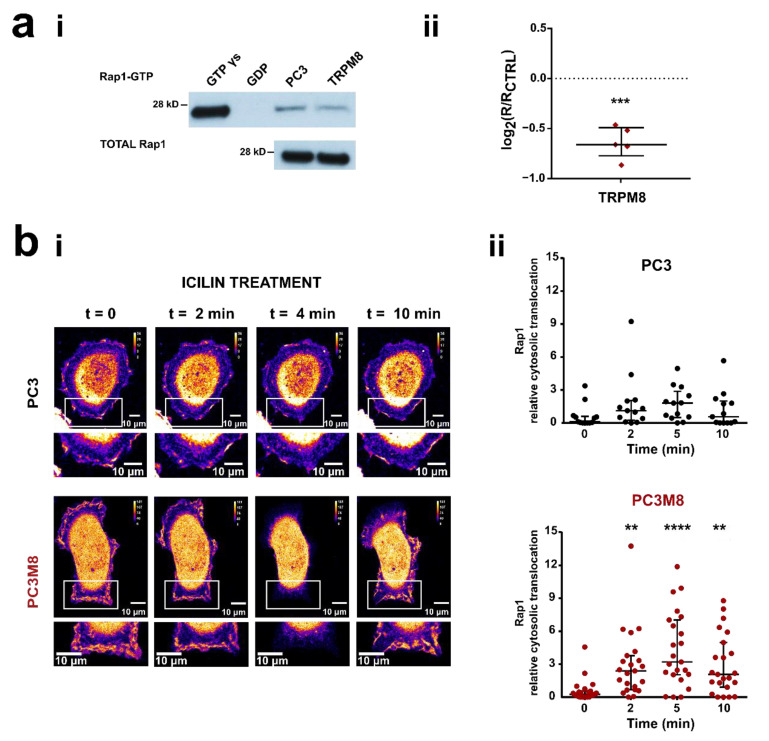
TRPM8 decreases Rap1 activation. (**ai**) Active Rap1 pull-down assay on PC3 and PC3 overexpressing TRPM8. The original uncropped blot is reported in Figure S2. (**aii**) Quantification of the active Rap1 as the ratio between Rap1-GTP over total Rap1 (R) normalized on this ratio on PC3 (R_CTRL_). Data are expressed as log_2_ and shown in the scatter dot plot in which each dot represents one single experiment and lines represent mean ± SEM. Statistical significance versus PC3, ***: *p* < 0.001 (One sample *t*-test). (**bi**) Live-cell imaging by confocal microscopy of PC3 and PC3M8 expressing GFP-RBD_RalGDS_ probe. Representative images showing GFP-RBD_RalGDS_ in PC3 and PC3M8 at *t* = 0 and after 2, 4 and 10 min of treatment with 10 μM icilin; the bottom parts of the figures represent enlargement of the inset (white box) for each time point. (**bii**) Scatter dot plots showing the quantification of GFP-RBD membrane recruitment calculated as cytosol translocation as described in the method section. Each dot represents one cell (83 regions of interest [ROIs] referring to 13 cells for PC3 and 100 ROIs referring to 23 cells for PC3M8) and lines show median ± interquartile range. Statistical significance versus *t* = 0, **: *p* < 0.005; ****: *p* < 0.0001 (Kruskal–Wallis test with Dunn’s *post-hoc* test).

Given the interaction between Rap1A and TRPM8 previously demonstrated [44,47], we further assessed whether Rap1 activation is involved in TRPM8-mediated inhibition of PC3 cell migration by using two distinct approaches. We first performed pull-down assays for GTP-bound Rap1, using GST-RalGDS, which binds active Rap1-GTP (Figure 2ai and Figure S2). PC3 cells were transfected with TRPM8 (4 μg) and stimulated with 10 μM icilin for 15 min. Interestingly, TRPM8 overexpression led to a decrease of 36% ± 13% of the amount of active Rap1-GTP protein as compared with control PC3 wt transfected with an empty vector (Figure 2aii). At the same time, TRPM8 overexpression does not affect the total amount of Rap1 (Figure 2aii and Figure S3).

The spatiotemporal activation of Rap1 was evaluated by live-cell GTPase activity assays, transfecting PC3 and PC3 stably overexpressing TRPM8 (PC3M8) with the GFP-RBD_RalGDS_ probe. This latter was developed to detect the intracellular spatial and temporal activation of Rap1, exploiting its high bond affinity in the active form (GTP-bounded) for the Ras binding domain (RBD) of the Rap1 effector RalGDS [60,61]. Since the active Rap1 pool localization is restricted to the PM, we considered the membrane recruitment of GFP-RBD as an indirect indication for Rap1 activation, as previously reported [47]. In particular, the cytosolic retention fraction of GFP-RBD upon icilin treatment was measured (see Materials and Methods). In PC3 wt GFP-RBD_RalGDS_ localization at the PM did not change upon icilin treatment (Figure 2bii on the top), indicating no changes in Rap1 activation. Conversely, in cells overexpressing TRPM8 the translocation of the active form of Rap1 to the PM was significantly inhibited upon icilin treatment, with a maximal effect on the cytoplasmic translocation 5 min after the treatment, and partial recovery after 10 min (Figure 2bii on the bottom).

**Figure 3:**
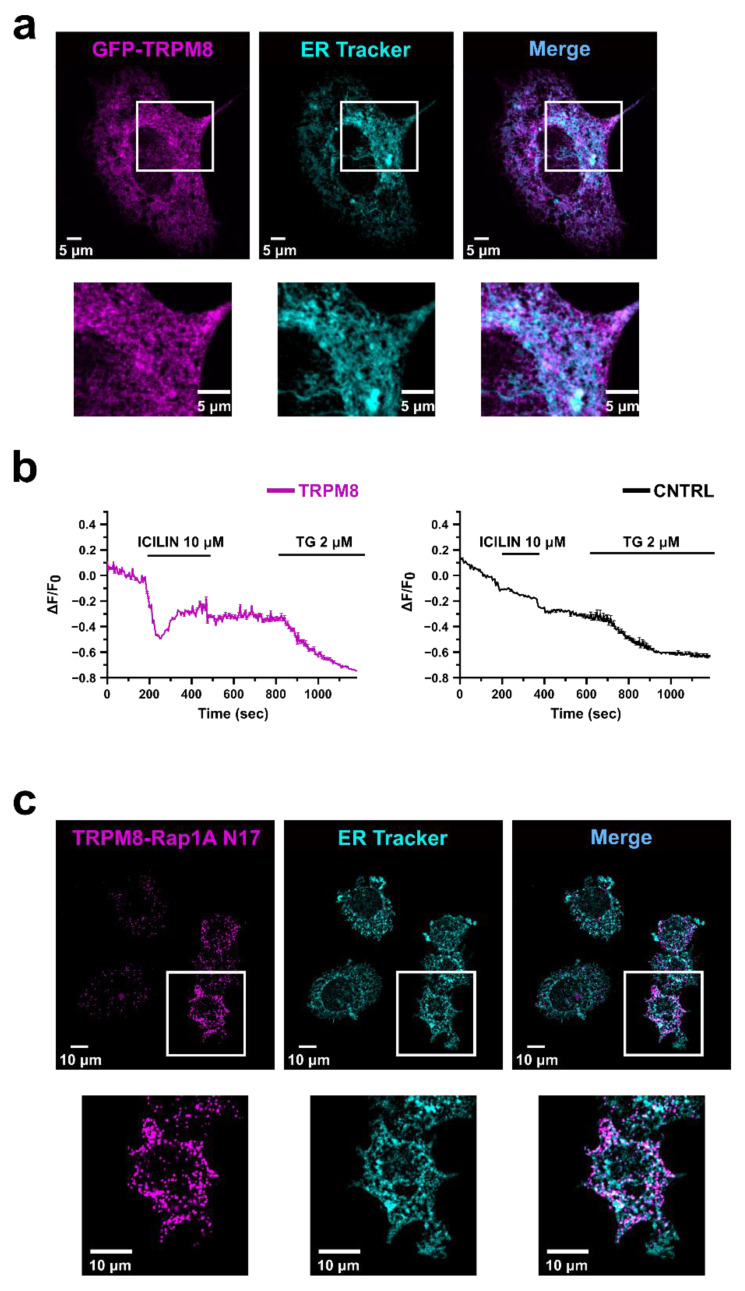
TRPM8 ER localization. (**a**) Representative confocal fluorescence images of PC3 cells 24 h after transfection with GFP-TRPM8. Magenta signal (on the left) refers to GFP-TRPM8 (excitation at 488 nm), cyan signal (in the center) refers to ER-tracker Red (excitation at 561 nm), and light blue in the merged image (on the right) indicates overlapped regions; below: zoom on a region of interest indicated by a white box. Scale bar: 5 μm. (**b**) ER Ca^2+^-imaging traces in response to TRPM8 agonist (10 µM icilin) and SERCA inhibitor (2 μM thapsigargin) in PC3 transiently overexpressing GFP-TRPM8 (magenta) and PC3 used as control (black). [Ca^2+^]_ER_ was visualized in PC3 cells expressing GEM-Cepia1er, a Ca^2+^ biosensor targeted to the ER. Traces represent the mean ΔF/F_0_ ± SEM from different ROIs within one representative cell for each condition (*n* = 7 for PC3, *n* = 3 out of 15 for TRPM8). (**c**) Representative confocal fluorescence images of PC3 cells 24 h after transfection with TRPM8 wt and Rap1A N17. Magenta signal (on the left) refers to TRPM8-Rap1A N17 interaction detected as fluorescent PLA dots, cyan signal (in the center) refers to Ds-Red ER probe (excitation at 561 nm), and light blue in merged image (on the right) indicates overlapped regions; below: zoom on a region of interest indicated by a white box. Scale bar: 10 μm.

The inhibitory role of TRPM8 on Rap1 activation is compatible with TRPM8 localization in the ER. Indeed, in the ER it could bind Rap1 in its inactive GDP-bound form, thus preventing the GTP exchange and the consequent translocation to the PM, as previously demonstrated for ECs, which, however, express the short isoform of TRPM8 on the ER [47]. On the other hand, when overexpressed in PC3, TRPM8 is located at least in part at the PM [43], favoring Ca^2+^ influx into the cell as shown in Figure S1. To test the hypothesis that, once overexpressed in PC3 cells, TRPM8 is also present and functional on the ER membranes, we performed confocal analysis on PC3 cells overexpressing the channel, showing a partial co-localization of TRPM8 with the ER staining (Figure 3a). Moreover, using a Ca^2+^ biosensor targeted to the ER (GEM-Cepia1er), we measured the activity of intracellular TRPM8. Stimulation of PC3 cells overexpressing TRPM8 with icilin (10 µM) induced a Ca^2+^ release from the ER stores in 20% of the cells tested, while no response was detected in CNTRL PC3 overexpressing the empty vector (Figure 3b). Finally, to localize the TRPM8-Rap1 interaction at the cellular level we performed proximity ligation assays on PC3 cells overexpressing both TRPM8 and the inactive form of Rap1 (Rap1 N17), which has been shown to bind TRPM8 in vitro [47]. As shown in Figure 3c, the protein interaction complexes indicated by fluorescent dots are partially localized on the ER, thus confirming the hypothesis that within the cell TRPM8 acts as a sponge by trapping intracellularly Rap1 in its inactive form, thus avoiding its translocation and activation on the PM.

### 3.3. Identification of the Residues Involved in TRPM8-Rap1 Interaction

**Figure 4:**
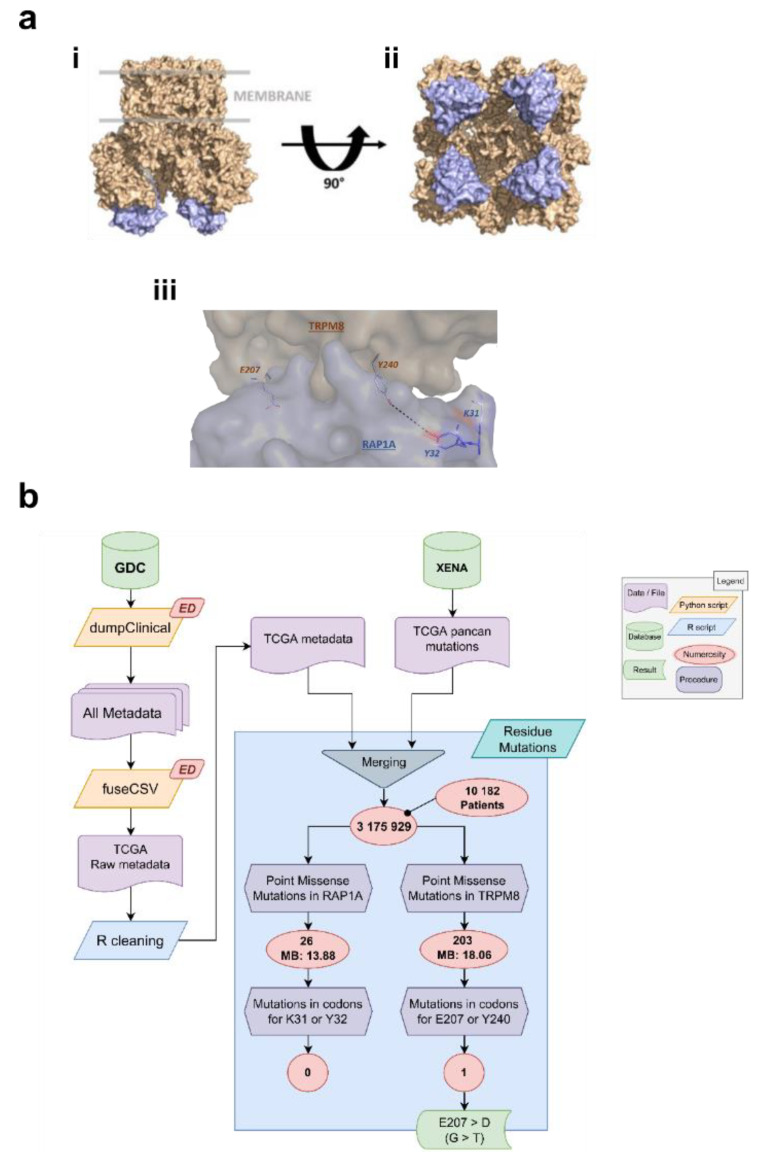
Identification of the residues involved in TRPM8-Rap1 interaction. (**ai**) Model of the interaction between TRPM8 (brown surface) and Rap1A (blue surface) obtained after docking simulation. (**aii**) 90° rotation view of the same model showing the tetrameric form of the TRPM8 receptor and the four interacting Rap1 proteins. (**aiii**) Focus on residues identified as hotspots after the residue centrality analysis performed on the best docking poses 3.4. (**b**) Workflow followed to investigate the presence of point mutations relative to residues identified by molecular modeling in real patients (*n* = 10,182).

Aiming to identify putative residues involved in the interaction between Rap1A and TRPM8, a molecular modeling approach has been used. First of all, as the C-tail was not solved for the TRPM8 structure, we modeled this tetrameric structure by homology modeling. This model was further used to predict the interaction between TRPM8 and Rap1A, for which a crystal structure existed (PDB ID: 4HDO). This prediction was obtained using a protein-protein docking algorithm and more specifically the ClusPro2.0 and InterevDock webservers. The docking was performed on one monomer of the TRPM8 structure, removing the trans-membrane part of the protein for the calculation. After docking poses were obtained, we superimposed all these poses onto the tetrameric structure of TRPM8 and we removed the poses that were not allowed due to unacceptable steric clashes. In the end, we obtained a model that was used to identify hotspots between Rap1A and TRPM8 (Figure 4a). For this purpose, a residue centrality analysis (RCA) and mapping of central residues was performed on the best-scored docking poses TRPM8 and Rap1A (Figure S4). Indeed, the two best docking poses of InterEvDock and three docking poses of ClusPro were used for residue interaction network generation followed by residue centrality analysis. Only residues at the docking interface were considered. A visual analysis of docking poses was considered and the maximum Z-score values from the residue centrality analyses performed on these docking poses were kept and displayed for four residues of interest, namely Glu207 and Tyr240 for TRPM8 and Lys31 and Tyr32 for Rap1A, respectively. These residues are displayed as sticks in the structure (Figure 4aiii) and proposed for site-directed mutagenesis. 

To deepen the clinical relevance of our findings we investigated the presence of point mutations on the key residues identified by molecular modeling in real patients. For this purpose, we used mutational data from the TCGA consortium. The full workflow and results of this analysis are shown in Figure 4b. The pan-cancer dataset included 3,175,929 point missense mutations from a global cohort of 10,182 patients. Of these, 26 mutations occurred on the *RAP1A* gene, and 203 on the *TRPM8* gene. The mutational burden (MB calculated as mutations per patient per megaresidue) of both genes was comparable, and, more precisely 13.88 and 18.06 for *RAP1A* and *TRPM8*, respectively. Filtering for mutations on the codons of residues E207/Y240 of the *TRPM8* gene, a single entry (from a single patient) emerged: a transversion on the codon for the Glutamic Acid (E) 207, causing it to become an Aspartic Acid (D). We found no mutations on the codons for K31/Y32 residues of the *RAP1A* gene.

### 3.4. Characterization of the Residues Involved in TRPM8-Rap1 Interaction

#### 3.4.1. Role of the Residue Y32 in the Interaction of GDP-Bound Rap1 with TRPM8 Cytosolic N-Terminal Tail

**Figure 5:**
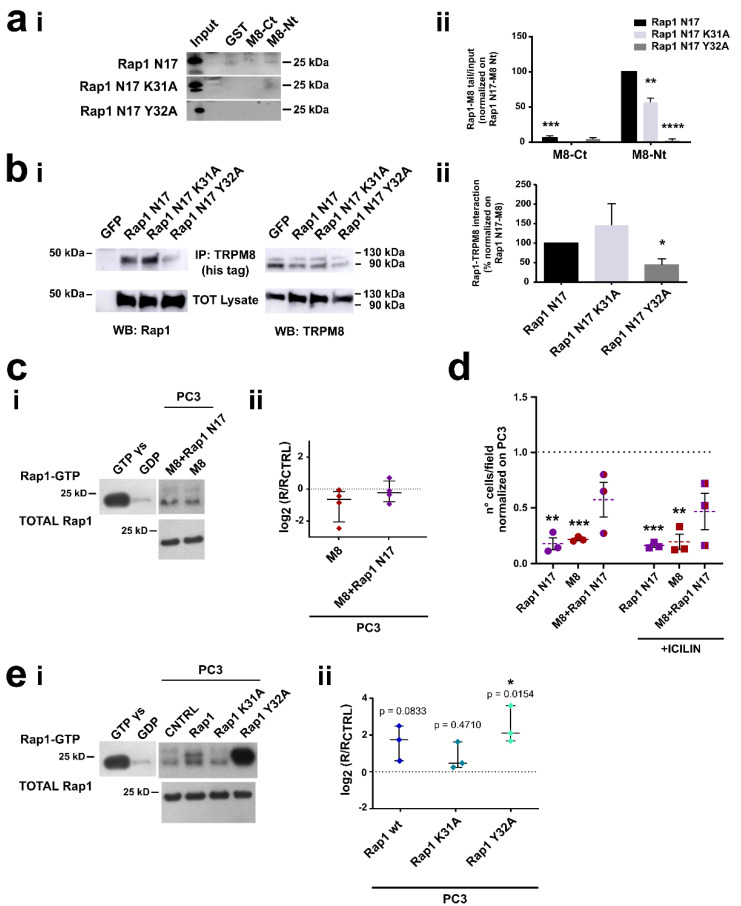
Rap1 mutants’ characterization. (**ai**) GST pull-down assay on TRPM8 N-terminal tail (M8-Nt), C-terminal tail (M8-Ct), or GST incubated with in vitro translated Rap1 N17, GFP-Rap1 N17 K31A and GFP-Rap1 N17 Y32A. 10% of the in vitro translated Rap1 N17 and mutants were used for the input of the GST pull-down. Western blotting was performed with an anti-Rap1 antibody and one representative experiment of three is shown. (**aii**) Quantification of Rap1-TRPM8 tails interaction normalized on the input. Values are normalized on Rap1 N17-M8Nt ratio and data are expressed as mean ± SEM. Statistical significance versus Rap1 N17 -TRPM8 Nt: **: *p* < 0.01; ***: *p* < 0.001; ****: *p* < 0.0001 (Two-way ANOVA with Tukey’s HSD *post-hoc* test). (**bi**) Representative immunoprecipitation experiment. PC3M8 cells were transfected with GFP-Rap1 N17, GFP-Rap1 N17 K31A and GFP-Rap1 N17 Y32A plasmids. Cell lysates were immunoprecipitated (IP) for TRPM8 and immunoblotted with anti-GFP antibody for Rap1 detection (panels on the left) and anti-myc antibody for TRPM8 detection (panels on the right). (**bii**) Quantification of Co-IP experiments on TRPM8 and Rap1 mutants. Data are normalized on TRPM8-Rap N17 interaction and they are expressed as mean ± SEM (*n* = 4). Statistical significance versus TRPM8-Rap1 N17, * = *p* < 0.05 (Student *t*-test). (**ci**) Active Rap1 pull-down assay on PC3 stably overexpressing TRPM8 transfected with Rap1 mutants (8 μg). (**cii**) Quantification of the active Rap1 as the ratio between Rap1-GTP over total Rap1 (R) normalized on this ratio on PC3 (R_CTRL_). Data are expressed as log_2_ and shown in a scatter dot plot in which each dot represents one single experiment and lines represent mean ± SEM. Statistical significance versus PC3 is represented by dotted line (RM one-way ANOVA without Geisser-Greenhouse correction and with Dunnett’s *post-hoc* test). (**d**) Quantification of adhesion assays on PC3 overexpressing Rap1 N17 (in violet) or TRPM8 (in red) or both Rap1 N17 and TRPM8 (ratio 1:1), treated or not with icilin (10 μM). Data are normalized on PC3 wt (dot line) and displayed as mean ± SEM of three independent experiments. Statistical significance vs. PC3 wt, **: *p* < 0.01; ***: *p* < 0.001 (One sample *t*-test). (**ei**) Active Rap1 pull-down assay on PC3 transfected with Rap1 mutants (8 μg). (**eii**) Quantification of the active Rap1 as the ratio between Rap1-GTP over total Rap1 (R) normalized on this ratio on PC3 (R_CTRL_). Data are expressed as log_2_ and shown in a scatter dot plot in which each dot represents one single experiment and lines represent mean ± SEM. Statistical significance versus PC3 (represented by dot line), *: *p* < 0.05 (RM one-way ANOVA without Geisser-Greenhouse correction and with Dunnett’s *post-hoc* test). All the original uncropped blots referring to this figure are reported in Figure S5.

As described above, molecular modeling experiments identified two putative critical amino acid residues on the Rap1 sequence for the Rap1-TRPM8 interaction: lysine (K) 31 and tyrosine (Y) 32 (Figure 4). We next validated these two putative interaction sites, assessing their effective involvement in Rap1-TRPM8 interaction as well as their functional consequences on protein activity and cellular responses, such as migration and adhesion. We started by investigating the role of K31 and Y32 residues in mediating Rap1 N17 interaction with TRPM8 wt. To do that, we produced and purified the TRPM8 N-terminal tail and C-terminal tail fused to GST and then we performed GST pull-down with in vitro translated Rap1 mutants (Figure 5a and Figure S5). Results showed that Rap1 N17 mainly directly interacts with the TRPM8 cytosolic N-terminal tail (Nt), in agreement with previous data [47], and indicated that both K31A and Y32A mutations significantly decreased Rap1 N17 interaction with TRPM8 Nt, with a total loss of the binding in the case of Y32A (Figure 5aii).

To further corroborate the contribution of these two residues on the binding of Rap1 N17 with TRPM8 and to validate it also in the cellular context, we generated both mutants by site-directed mutagenesis and assessed their interaction by co-immunoprecipitation assay. PC3M8 cells were transfected with GFP-Rap1 N17 K31A or GFP-Rap1 N17 Y32A mutants and cell lysates were immunoprecipitated for TRPM8 (his-tagged) and then immunoblotted with anti-GFP antibody for Rap1 detection and anti-myc antibody for TRPM8 detection (Figure 5bi). We show that TRPM8 and Rap1 N17 interact in intact prostate cancer cells, validating docking analyses since the point mutation of Y32 in the Rap1 sequence significantly weakened the intracellular interaction with TRPM8 (Figure 5bii). 

To further validate the hypothesis that TRPM8 traps the inactive form of Rap1 and thus prevents its activation on the PM, we investigated the functional effects of Rap1 N17 mutant, well-defined in literature as Rap1 dominant-negative due to its ability to significantly reduce GEF activity rate and thereby substantially block Rap1 in its inactive (GDP-bound) form [69]. According to our hypothesis about TRPM8 acting as a sponge for inactive Rap1, pull-down assays for active (GTP-bound) Rap1 showed that in cells overexpressing TRPM8 in the presence of an excess of inactive Rap1 (i.e., overexpressing Rap1 N17) the amount of active Rap1 is higher, although not significantly, with respect to cells overexpressing TRPM8 in the absence of an excess of inactive Rap1 (Figure 5c). Consistently, TRPM8 and Rap1 N17 co-overexpression reverted the TRPM8-mediated inhibition of PC3 adhesion (Figure 5d). More in detail, in the presence and in the absence of icilin we observed a significant inhibition of cell adhesion both in cells that overexpress TRPM8 alone as previously observed (Figure 1d and Figure 5d) and in cells that overexpress only Rap1 N17. According to the role of Rap1 N17 as dominant-negative, the above inhibition can be explained by overloading the cell with Rap1 in its inactive form. However, the overexpression of both TRPM8 and Rap1 N17 did not affect PC3 cells adhesion both in the presence and in the absence of icilin (Figure 5d), confirming the tight relationship between the channel and the inactive (GDP-bound) form of Rap1 in mediating cell adhesion independently from the channel activity. 

Next, before functionally validating the involvement of K31 and Y32 in the Rap1 sequence in mediating the interaction with the TRPM8 sequence, we evaluated their possible effects on Rap1 activation (Figure 5e). Interestingly, Rap1 Y32 mutant significantly increased Rap1 activation in PC3 cells (Rap1 Y32 vs. CNTRL: * *p* < 0.05), whereas Rap1 wt and Rap1 K31 mutant did not alter it (Rap1 wt vs. CNTRL: *p*-value = 0.0833; Rap1 K31 vs. CNTRL: *p*-value = 0.4710). This result could suggest an intrinsic effect of Y32 on Rap1 activation itself even in the absence of TRPM8, and therefore, we did not go further in the functional validation of the Rap1 Y32A mutant, as its possible involvement in the interaction with TRPM8 could be altered or masked from an intrinsic activity of the residue on Rap1 properties.

#### 3.4.2. Role of Residues E207 and Y240 in the Interaction of TRPM8 Cytosolic N-Terminal Tail with GDP-Bound Rap1

**Figure 6:**
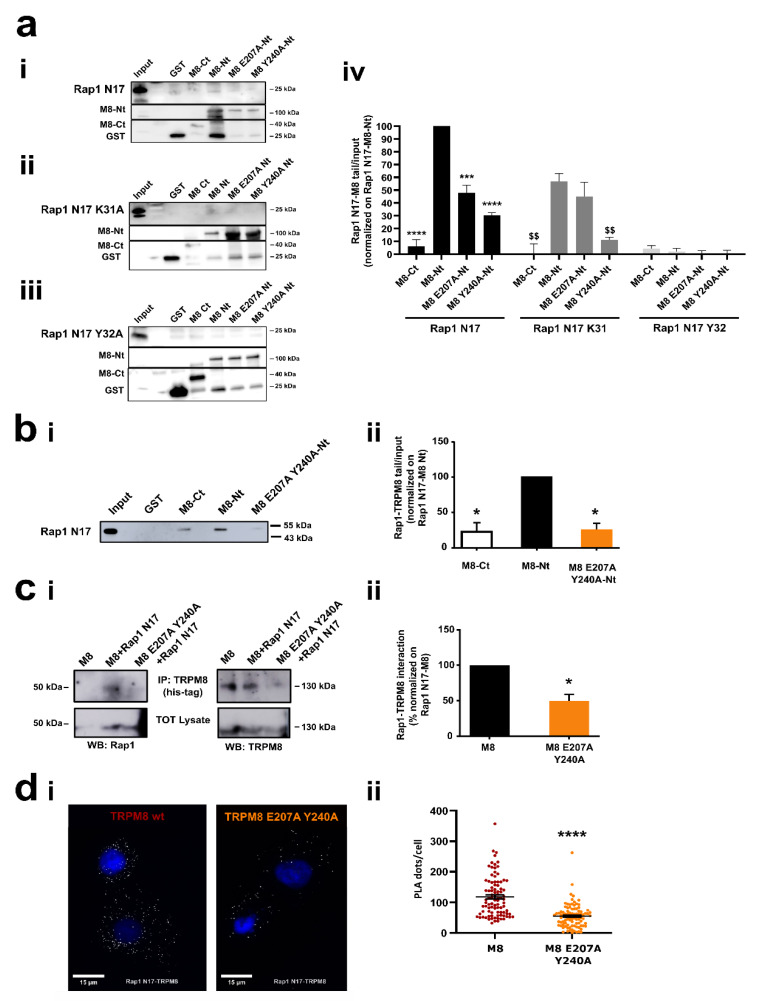
TRPM8 mutants’ characterization. (**a**) GST pull-down assay on GST, TRPM8 wt C-terminal tail (M8-Ct), TRPM8 wt N-terminal tail (M8-Nt), TRPM8 E207A N-terminal (M8 E207A-Nt) and TRPM8 Y240A N-terminal (M8 Y240A-Nt), incubated with in vitro translated Rap1 N17 (**ai**), Rap1 N17 K31A (**aii**) and Rap1 N17 Y32A (**aiii**). 5% of the lysates were used for the input of the GST pull-down. Western blotting was performed with an anti-Rap1 antibody and one representative experiment out of three is shown. The original uncropped blots are reported in Figure S5. (**aiv**) Quantification of Rap1 N17-TRPM8 tails interaction normalized on the input. Values are normalized on Rap1 N17-M8-Nt ratio and data are expressed as mean ± SEM. Statistical significance versus Rap1 N17-TRPM8-Nt, ***: *p* < 0.001; ****: *p* < 0.0001; statistical significance versus Rap1 N17 K31-TRPM8-Nt, $$: *p* < 0.01 (Student’s *t*-test). (**bi**) GST pull-down assay on GST, M8-Ct, M8-Nt and M8 E207A Y240A-Nt, incubated with lysates of PC3 cells overexpressing GFP-Rap1 N17. 5% of the lysates were used for the input of the GST pull-down. Western blotting was performed with anti–GFP antibody and one representative experiment of three is shown. (**bii**) Quantification of Rap1 N17-TRPM8 tails interaction normalized on the input. Values are normalized on Rap1 N17-M8 wt-Nt ratio and data are expressed as mean ± SEM (*n* = 3). Statistical significance versus Rap1 N17-TRPM8 wt-Nt, *: *p* < 0.05 (paired Student’s *t*-test). (**ci**) Representative immunoprecipitation experiment. PC3 cells were transfected with GFP-Rap1 N17 and TRPM8 wt/E207A Y240A plasmid. Cell lysates were immunoprecipitated (IP) for TRPM8 (his-tagged) and immunoblotted with anti-GFP antibody for Rap1 detection (panels on the left) and anti-TRPM8 antibody for TRPM8 detection (panels on the right). (**cii**) Quantification of Co-IP experiments on TRPM8 and Rap1 N17. Data are normalized on Rap1 N17-TRPM8 (wt) interaction and they are expressed as mean ± SEM (*n* = 4). Statistical significance versus Rap1 N17-TRPM8 (wt), *: *p* < 0.05 (paired Student *t*-test). (**di**) In situ detection of TRPM8/Rap1 interaction in PC3 cells overexpressing TRPM8 (wt or E207A Y240A) and Rap1 N17. TRPM8/Rap1 complexes were monitored as red fluorescent dots using PLA and cell nuclei are visualized in blue (DAPI staining). (**dii**) Puncta density quantified as mean ± SEM of puncta per cell from three independent experiments (n= 92 for both M8 and M8 E207A Y240A). Statistical significance versus TRPM8 wt, ****: *p* ≤ 0.0001 (Mann-Whitney test). The original uncropped blots referring to panels b and c are reported in Figure S6.

After the study and the characterization of Rap1 residues involved in the binding with TRPM8, we focused on TRPM8 residues predicted to be critical for the interaction. Molecular modeling analyses identified two amino acid residues in the TRPM8 sequence that may be involved in the binding with Rap1: glutamate (E) 207 and tyrosine (Y) 240 (Figure 4). To evaluate the contribution of these two residues on the binding of TRPM8 with inactive Rap1 N17 mutant we performed GST pull-down assays between GST-TRPM8 Ct, GST-TRPM8 Nt, GST-TRPM8 E207A, and GST-TRPM8 Y240A Nt fragments with in vitro translated Rap1 N17 (Figure 6ai), Rap1 N17 K31A (Figure 6aii) and Rap1 N17 Y32A (Figure 6aiii). Indeed, both E207A and Y240A mutations significantly reduced the interaction between TRPM8 Nt wt and Rap1 N17 (Figure 6a, * symbols). Moreover, our data showed that this reduction is even more pronounced in the presence of Rap1 N17 Y32A (Figure 6a, $ symbols), whereas no differences were observed between TRPM8 mutants-Rap1 N17 and TRPM8 mutants-Rap1 K31A interactions. K31 in Rap1 N17 sequence revealed an effect just in the interaction with TRPM8 Nt wt (Figure 6a, # symbols). Overall, these data strongly validated molecular modeling predictions, confirming that all the mutations combined (E207A Y240 on TRPM8 sequence and Y32A on Rap1 N17 sequence) led to a complete loss of TRPM8-Rap1 interaction (Figure 6aiv). Since the contribution given by TRPM8 mutants on the interaction with Rap1 N17 was comparable, we decided to generate a single TRPM8 double mutant, carrying both the mutations (TRPM8 E207A Y240A) and we validated it through both GST-pull down (Figure 6b) and Co-IP experiments (Figure 6c). As shown in Figure 6b,c, the TRPM8 double mutant still inhibits the interaction between TRPM8 Nt cytosolic tail and the inactive Rap1 N17. Moreover, we further validated this finding through proximity ligation assay (PLA), showing that in the presence of TRPM8 E207A Y240A the number of dots representing TRPM8-Rap1 complexes is significantly reduced in comparison with TRPM8 wt (Figure 6d).

### 3.5. TRPM8 E207 Y240 Residues Revert Rap1-Mediated Inhibition of Cancer Cell Adhesion and Migration

**Figure 7:**
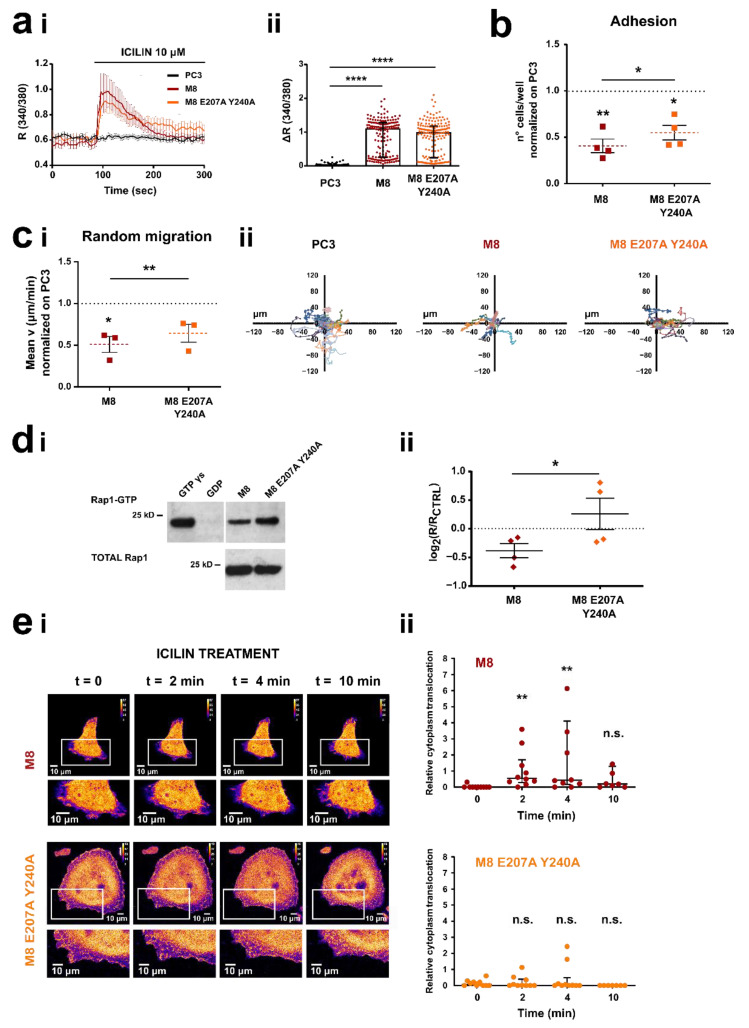
TRPM8 mutant’s functional validation. (**ai**) Ca^2+^-imaging traces in response to TRPM8 agonist (10 µM icilin) in PC3 (control in black), PC3 transiently overexpressing TRPM8 (red) and PC3 transiently overexpressing TRPM8 E207A Y240A mutant (orange). Traces represent the mean ± SEM of cells in the recorded field of one representative experiment (*n* = 45 for PC3, *n* = 32 for M8 and *n* = 33 for M8 E207A Y240A). (**aii**) Scatter dot plot showing peak amplitude of icilin-mediated Ca^2+^ responses (median with interquartile range of different cells in the field from at least 7 independent experiments). Only icilin-responsive cells were considered to compare TRPM8 and TRPM8 E207A Y240A channel activity (*n* = 228 for PC3, *n* = 160 for M8 and *n* = 167 for M8 E207A Y240A). Statistical significance versus PC3, ****: *p* < 0.0001 (Kruskal–Wallis test with Dunn’s *post-hoc* test). (**b**) Quantification of adhesion assays on PC3 overexpressing TRPM8 wt (red) or TRPM8 E207A Y240A (orange) and treated with icilin (10 μM). Data are normalized on PC3 treated with icilin (control represented by dot line) and displayed as mean ± SEM of four independent experiments. Statistical significance versus PC3, *: *p* < 0.05; **: *p* < 0.01 (One sample *t*-test); statistical significance M8 wt vs. M8 E207A Y240A, *: *p* < 0.05 (paired Student *t*-test). (**ci**) Quantification of random migration assays on PC3 overexpressing TRPM8 wt (red) and PC3 overexpressing TRPM8 E207A Y240A mutant (orange) in the presence of icilin (10 µM). Data are normalized on PC3 treated with icilin (control represented by dot line) and displayed as mean ± SEM of three independent experiments. Statistical significance versus PC3 when not differently specified, *: *p* < 0.05; **: *p* < 0.01 (One sample *t*-test); statistical significance M8 wt vs. M8 E207A Y240A, *: *p* < 0.05 (paired Student *t*-test). (**cii**) Representative migration plots of PC3 (left panel), PC3 overexpressing TRPM8 (central panel) and PC3 overexpressing TRPM8 E207A Y240A (right panel). Each line represents the trajectory of one cell within a 6 h period. Data shown refer to one field of one representative experiment out of three (PC3 = 20 cells; M8 = 16 cells; M8 E207A Y240A = 17 cells). (**di**) Active Rap1 pull-down assay on PC3 overexpressing TRPM8 wt and TRPM8 E207A Y240A (4 μg). The original uncropped blot is reported in Supplimentary Figure S6. (**dii**) Quantification of the active Rap1 as the ratio between Rap1-GTP over total Rap1 (R) normalized on this ratio on PC3 (R_CTRL_). Data are expressed as log_2_ and shown in a scatter dot plot in which each dot represents one single experiment and lines represent mean ± SEM. Statistical significance *: *p* < 0.05 (paired Student’s *t*-test). (**ei**) Live-cell imaging by confocal microscopy of PC3 overexpressing TRPM8 wt or TRPM8 E207A Y240A and expressing GFP-RBD_RalGDS_ probe. Representative images showing GFP-RBD_RalGDS_ in PC3 + TRPM8 and PC3+ TRPM8 E207AY240A at *t* = 0 and after 2, 5 and 10 min of treatment with 10 μM icilin; the bottom parts of the figures represent an enlargement of the inset (white box) for each time point. (**eii**) Scatter dot plots showing the quantification of GFP-RBD membrane recruitment calculated as cytosol translocation as described in the method section. Each dot represents one cell (51 ROIs referring to 10 cells for TRPM8 and 57 ROIs referring to 10 cells and for TRPM8 E207A Y240A) and lines show the median ± interquartile range. Statistical significance versus *t* = 0, **: *p* < 0.005 (Kruskal–Wallis test with Dunn’s *post-hoc* test).

Given the validation of the TRPM8 double mutant showing that E207 and Y240 residues are key players in the TRPM8-Rap1A interaction, we functionally validated the interaction. We first characterized the activity of TRPM8 E207A Y240A as a functional ion channel. Ca^2+^ imaging experiments clearly showed that icilin (10 µΜ) stimulation promotes an increase in [Ca^2+^]_i_ in both PC3 overexpressing TRPM8 wt or TRPM8 E207A Y240A as compared with PC3 transfected with empty plasmid (Figure 7a), demonstrating that mutations did not alter channel activity (not significance between TRPM8 and TRPM8 E207A Y240A—Figure 7aii). 

Next, to validate the functional role of E207A Y240A mutations we performed adhesion assays on PC3 overexpressing TRPM8 wt or TRPM8 E207A Y240A in the presence of icilin (10 µM) (Figure 7b). The results showed that TRPM8 double mutant significantly reverts the inhibition of PC3 cell adhesion induced by TRPM8 wt (Figure 7b). More specifically, in the presence of icilin cell adhesion is inhibited on average by 59% by TRPM8 wt and by 45% by TRPM8 E207A Y240A. Similar results were also obtained by random migration assay, showing that under icilin stimulation (10 µM) TRPM8 E207A Y240 does not affect PC3 cell migration speed revealing a significant difference compared to TRPM8 wt (Figure 7c). Aiming to ascertain that this lower inhibitory effect exhibited by the double mutant on migration and adhesion underlies a less marked inhibition on Rap1 activation, we performed active Rap1 pull-down and live-cell imaging experiments on PC3 cells overexpressing TRPM8 wt or TRPM8 E207A Y240A. Consistent with our hypothesis, E207A Y240A mutations, by reducing the interaction of TRPM8 with Rap1, resulted in less inhibition of the small GTPase activity: the amount of endogenous active (GTP-bound) Rap1 is significantly higher in the presence of TRPM8 double mutant as compared with TRPM8 wt (Figure 7d, TRPM8 wt vs. TRPM8 E207A Y240A: *, *p* < 0.05). Furthermore, after stimulation with icilin (10 µM), no significant intracellular retention of active Rap1 was mediated by the double mutant (Figure 7e on the bottom). On the contrary, TRPM8 wt showed a significant relative cytoplasmic translocation of active Rap1 (corresponding to an inhibition of its translocation to the PM, as previously described) already 2 min after treatment, which disappears after 10 min (Figure 7e on the top) in agreement with the results previously obtained (Figure 2b).

### 3.6. TRPM8-Rap1 Interaction in Breast and Cervical Cancer

**Figure 8:**
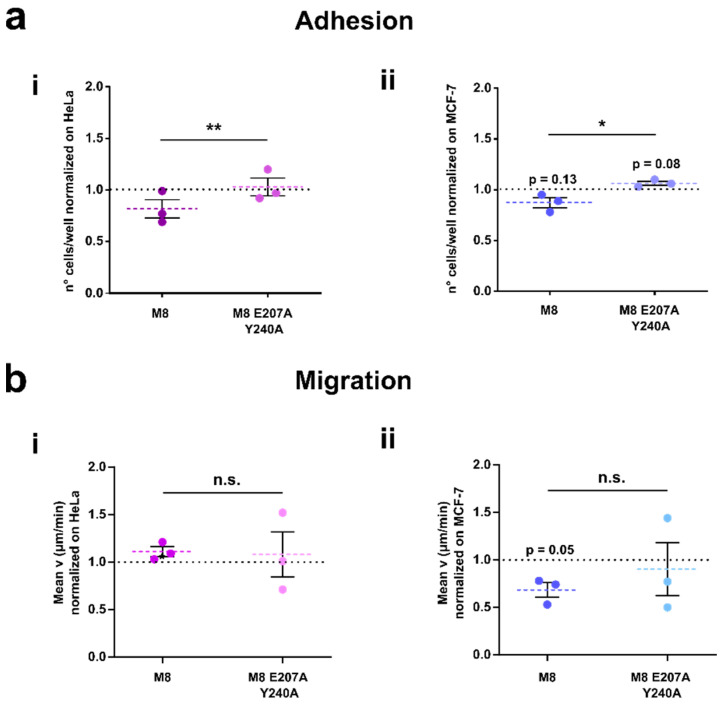
Validation of TRPM8 E207A Y240A on other epithelial cancer cell lines. (**a**) Quantification of adhesion assays on HeLa (**i**) and MCF-7 (**ii**) cells transfected with TRPM8 wt and TRPM8 E207A Y240A. Data are normalized on control cells (HeLa or MCF-7 cell transfected with empty vector represented by dot lines) and displayed as mean ± SEM of three independent experiments. Statistical significance versus HeLa/MCF-7 wt, *: *p* < 0.05; **: *p* < 0.01; n.s.: not significant (One sample *t*-test); statistical significance M8 wt vs. M8 E207A Y240A, *: *p* < 0.05; **: *p* < 0.01 (paired Student *t*-test). (**b**) Quantification of transwell migration assays on HeLa (**i**) and MCF-7 (**ii**) cells transfected with TRPM8 wt and TRPM8 E207A Y240A. Data are normalized on control cells (dot lines) and displayed as mean ± SEM of three independent experiments. Statistical significance versus HeLa/MCF-7 wt, n.s.: not significant (One sample *t*-test); statistical significance M8 wt vs. M8 E207A Y240A, n.s.: not significant (paired Student *t*-test).

A final set of experiments was performed to evaluate whether the mechanistic model illustrated here on the TRPM8-Rap1 interaction could potentially be generalized beyond prostate cancer. For this purpose, we assessed the TRPM8 double mutant on two other tumor cell lines (Figure 8) that do not express TRPM8 endogenously (Figure S3a). We performed adhesion and migration assays on HeLa cells and MCF-7 cells overexpressing TRPM8 wt or TRPM8 E207A Y240A. The TRPM8 transfection was confirmed by Western blot (Figure S3ai), and Ca^2+^ imaging analyses (Figure S3b). As shown in Figure 8a, both HeLa (i) and MCF-7 (ii) cells overexpressing TRPM8 E207A Y240A displayed a significant increase in cell adhesion compared to cells transfected with TRPM8 wt (Figure 8ai), M8 E207A Y240A vs wt in HeLa: **, *p* < 0.01; Figure 8aii M8 E207A Y240A vs wt in MCF-7: *, *p* < 0.05). As regards cell migration, we observed that in HeLa cells this pathway is not affected by either TRPM8 wt or TRPM8 E207A Y240A (Figure 8bi). Conversely, TRPM8 wt decreases cell migration in MCF-7 cells, while TRPM8 E207A Y240A does not (Figure 8bii), thus suggesting the involvement of the TRPM8-Rap1 interaction in the control of the migratory phenotype of breast cancer similarly to prostate cancer (Figure 7c). Furthermore, we have verified that this effect is not attributable to reduced cell viability. Indeed, we found that, in correspondence with the greatest overexpression of TRPM8, i.e., 24 h after transfection (Figure S7b), cell viability is not affected in MCF-7 (Figure S7aii) or in PC3 (Figure S7ai). However, it is interesting to note that disruption of the interaction between TRPM8 and Rap1 mildly reduces the viability of MCF-7 and PC3 (by 7% and 16%, respectively), thus suggesting a possible role of TRPM8-Rap1 interaction in preserving the viability of these two cell lines. In contrast, in HeLa cells, which did not show any TRPM8-mediated inhibition of cell migration, both TRPM8 wt and TRPM8 E207A Y240A showed a slight but significant reduction in cell viability of approximately 14 (Figure S7aiii).

**Figure 9:**
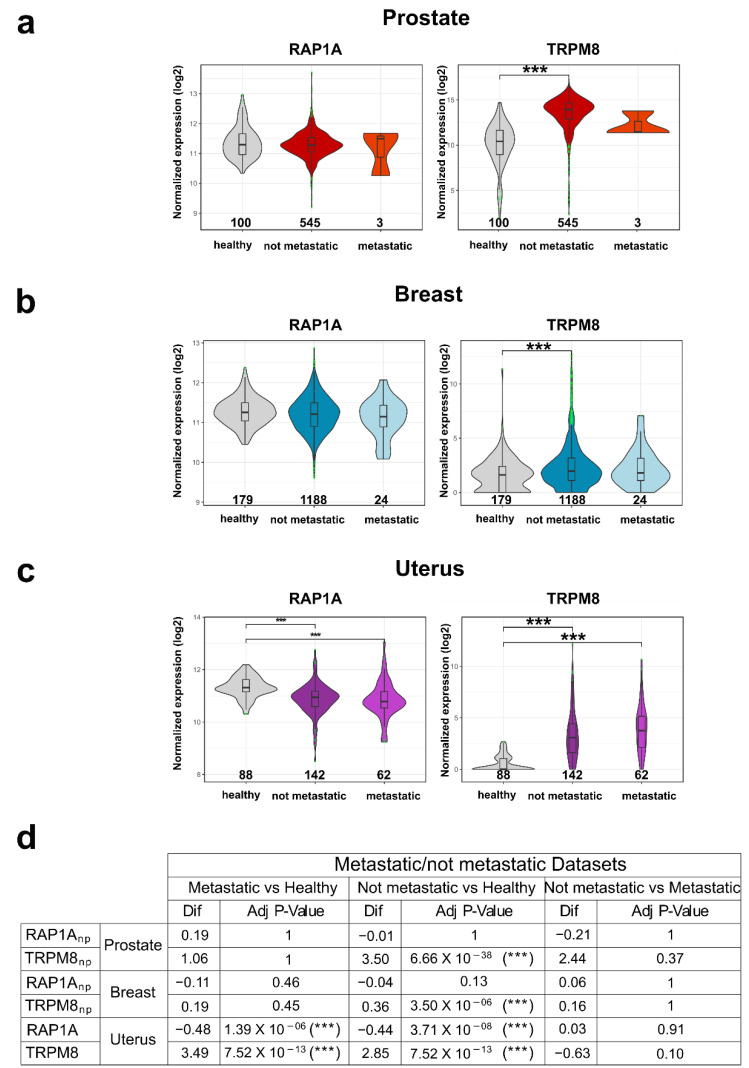
TRPM8 and Rap1A expression during carcinogenesis in different cancers. Differential expression analysis on Rap1A and TRPM8 transcripts in prostate (**a**), breast (**b**), and uterine (**c**) healthy and tumor (not metastatic and metastatic) patient samples from GTEx and TCGA databases, respectively. Results of statistical analysis are reported in the table of panel (**d**). Dif = difference between means; np = nonparametric hypothesis tests (i.e., Kruskal–Wallis H omnibus test and Dunn’s *post-hoc* test). Otherwise, ANOVA and Tukey’s HSD *post-hoc* test were performed; statistical significance ***: *p* < 0.001.

Finally, in order to investigate the clinical relevance of TRPM8-Rap1A relative expression in the cancer types for which we studied protein-protein interaction, we used transcriptional data from TCGA and GTEx databases to perform differential expression analysis on both genes. Specifically, we downloaded RNA-Seq data for prostate, breast, and uterine epithelia labeling samples as “healthy”, “metastatic” or “not metastatic” based on the clinical criteria described in the Materials and Methods section. Then, differential expression analysis was separately performed on *RAP1A* and *TRPM8* transcript levels in each one of the three cancer cohorts. As shown in Figure 9, TRPM8 expression increases in the three types of cancer in the “not metastatic” condition compared to healthy samples. The same can be observed when metastatic samples are compared to healthy controls in the uterine cohort but not in the breast and prostate ones. On the other hand, Rap1A expression remains almost unchanged in prostate and breast cancer while it decreases in uterine cancer (in both “metastatic” and “not metastatic” groups). Though the sample size of the “metastatic” group in the prostate cohort is actually too small (*n* = 3) to be really informative about the actual transcriptional profile associated with this condition, these results seem to suggest that prostate and breast cancers both share the same expression profile for *RAP1A* and *TRPM8* genes, in contrast to uterus cohort that showed instead a markedly different transcriptional pattern. Similarly, the cervical cancer cell line was the only in vitro model for which overexpression of TRPM8 failed to reduce migration while instead more markedly affecting cell viability in our experiments, further confirming the functional implications of *TRPM8-RAP1A* relative expression.

## 4. Discussion

The anti-metastatic role of TRPM8 in prostate cancer due to its ability to impair PCa cells’ motility has been suggested in recent years [43,44]. These findings are consistent with an androgen-dependency of TRPM8 expression which results in strong TRPM8 down-regulation during PCa progression [40,41,69]. Furthermore, it has previously been shown that TRPM8 is also expressed in different ECs and that it is dramatically down-regulated in some tumor-derived ECs, contributing to their more aggressive and pro-angiogenic migratory phenotype [47]. Our data confirmed the key role played by TRPM8 in inhibiting metastatic PCa cells’ migration (Figure 1c) and also revealed a role of the channel in the regulation of PC3 cell adhesion (Figure 1a). Consistent with data obtained in ECs [47], TRPM8 seems to exert its inhibitory effect on PCa cells’ migration independently from its channel function (Figure 1b,e). However, a role for Ca^2+^ influx seems to be important for the adhesion process as demonstrated by the lack of inhibitory effect on cells overexpressing TRPM8 pore mutant (Y905A) stimulated with TRPM8 agonist (Figure 1d,e). On the contrary, the migratory features of PC3 cells transfected with both TRPM8 wt and TRPM8 Y905A did not show any differences in the presence of icilin, supporting the hypothesis that TRPM8 effects on cell migration are completely independent of its channel function, as previously demonstrated for ECs [47]. Importantly, we revealed that TRPM8 expression is sufficient to exert its functional effects, inhibiting PC3-extracellular matrix adhesion and PC3 migration, although TRPM8 active stimulation may improve its inhibitory impact on cell adhesion probably through a Ca^2+^-dependent additive mechanism. Our results are consistent with emerging evidence for pore-independent roles of ion channels [70,71] including TRP channels in many hallmarks of cancer. In particular, the modulation of various intracellular pathways has previously been traced back to the enzymatic properties of some TRP channels [72,73] or to their coupling with other partner proteins that do not necessarily involve their pore activity [13,47,74]. 

In this regard, among different interactors of TRPM8 [44], our attention was focused on the small GTPase Rap1A, whose central role in the promotion of cell adhesion through the activation of the β_1_-integrin signaling pathway is well-known [75,76]. The interaction between TRPM8 and Rap1 was further supported by the partial punctate co-localization outside the nucleus of the two proteins shown in prostate clinical samples in Supplementary Figure S8. In the last decade, several studies have reported a strong interplay between TRP channels and small GTPases in the modulation of the metastatic cascade [21]. More specifically, TRP channels were found to affect small GTPase activity via both Ca^2+^-dependent and Ca^2+^–independent pathways. Conversely, small GTPases may act as TRP channel effectors as well as regulators, by modulating channel trafficking, gene expression, protein-protein interactions (PPIs), and channel gating [21]. Taken together, our results on Rap1 activation clearly show that, as observed in endothelial cells [47], TRPM8 overexpression and its activation by icilin in PC3 cells lead to the intracellular retention of Rap1 in its inactive (GDP-bound) form, decreasing the active (GTP-bound) Rap1 fraction available for the translocation to the PM (Figure 2). We then confirmed that also in intact PC3 cells the TRPM8-mediated inhibition on Rap1 activation is due to physical direct interaction between the channel and the inactive form of Rap1A (Figure 3c and Figure 5a,b). GST pull-down on in vitro translated Rap1 proteins had previously shown that the TRPM8 N-terminal tail mostly interacts with the inactive (GDP-bound) form of Rap1 (Rap1 N17 mutant) compared to Rap1 wt and its active form (Rap1 V12 mutant) [47]. This interaction was further confirmed in the cellular context in PC3 cells (Figure 3c and Figure 5b). These results are in agreement with our previous data on vascular endothelium, which showed that this coupling affects ECs migration, in vitro sprouting and vascular network formation [47].

**Figure 10:**
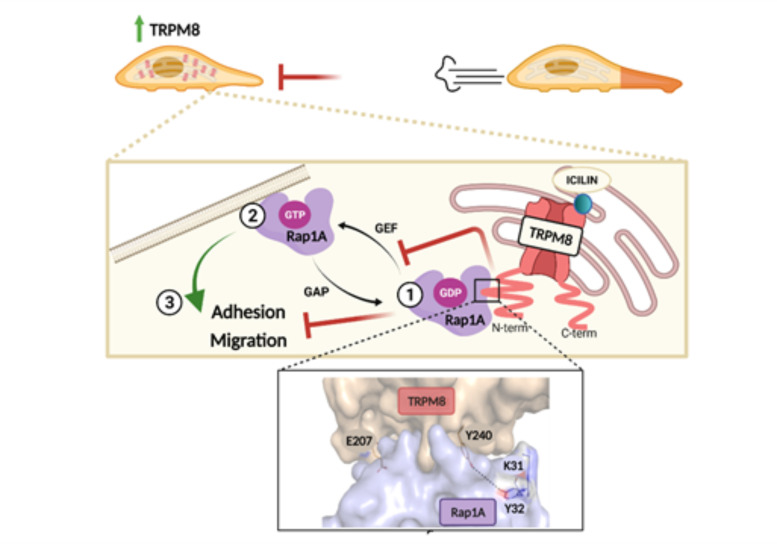
Scheme summarizing the mechanism through which TRPM8 inhibits cell adhesion and migration thanks to the direct interaction with the small GTPase Rap1A.

Our hypothesis on the intracellular trapping of Rap1 in its inactive form by TRPM8 is further corroborated by our data on the subcellular localization of TRPM8-Rap1A interaction. Small GTPases are known to exert their effects on cellular behavior mainly through a rigorous spatiotemporal regulation to which they and their effectors/modulators are subjected [77,78,79]. In particular, Rap1A in its active form is present at the PM, where its activation occurs allowing the β_1_-integrin signaling induction [76,80,81], whereas in its nucleotide-free form it is present in cytoplasmic vesicles, but not at the PM [60]. Therefore, the retention of inactive Rap1 would be more consistent with its intracellular localization in close proximity to both TRPM8 and ER. Our analysis of TRPM8-Rap1A N17 complexes revealed a partial co-localization with PC3 ER membrane (Figure 3c), in agreement with previous results obtained in ECs [47]. In PCa a dual localization of TRPM8 long isoform (130 kD), both on the PM (TRPM8_PM_) and the ER (TRPM8_ER_) has been extensively highlighted. This dual localization in the prostate not only is determined by the epithelial cell phenotype and by the androgen status [41,69] but has been also associated with a different channel function in carcinogenesis events, such as proliferation, apoptosis, and migration. More specifically, it has been shown that TRPM8_ER_ can be considered an important factor in controlling the apoptosis of advanced PCa metastatic cells [82,83], being capable of influencing the filling of ER stores [84], strictly related to the apoptosis-resistant cell phenotypes, characteristic of advanced prostate cancer [82,85,86]. Our results show that the TRPM8_ER_ fraction plays a role in PCa cells’ motility, similarly to ECs [47]. On the other hand, this would not exclude a possible Ca^2+^-mediated involvement of the TRPM8_PM_ fraction previously suggested [43]. Overall, we propose that the long TRPM8_ER_ isoform, beyond its Ca^2+^-dependent impact on cell growth and apoptosis, is also involved in the regulation of PCa migration and adhesion due to its interaction with the small GTPase Rap1A (Figure 10). The mechanism depicted has been further validated by the results obtained through active Rap1 pull-down and adhesion assays performed on PC3 cells overexpressing TRPM8 and the inactive GDP-bound Rap1 N17 mutant. Indeed, the inhibition induced by TRPM8 on Rap1 activation, as well as that on PC3 adhesion, partially reverted in the presence of overexpressed Rap1 N17 (Figure 5c,d). This partial functional reversion could be explained by the saturation of TRPM8 binding sites borne by the excess of inactive Rap1: due to the binding of TRPM8 with Rap1 N17 mutant, the amount of endogenous Rap1 available for the functional switch into the active GTP-bound form free to accomplish its function, promoting cell adhesion, is greater. Consequently, in systems overexpressing both TRPM8 and inactive Rap1, the inhibitory effect on Rap1 activation and cell adhesion is partially reverted. Notably, the TRPM8-Rap1A interplay shown here is not the only example of TRPM8 interaction with an inactive GTPase. Indeed, a direct interaction between TRPM8 and the G-protein subunit Gα_q_ even when inactive has been previously described, which ultimately results in the inhibition of TRPM8 gating [87]. The menthol-mediated activation of TRPM8 was found to exert a metabotropic activity through the functional and structural interaction with Gα_q_ [88]. Overall, there is a close bidirectional interaction between the TRPM8 channel and GTPase proteins supported by direct physical interactions: depending on the case, TRPM8 can act as both the modulator and effector of this functional coupling. 

A major discovery of our study is the identification of the residues involved in the interaction between TRPM8 and Rap1A. These findings could, indeed, give new insights for potential future applications of TRPM8 as therapeutic targets in PCa treatment. Among the two putative residues identified by molecular modeling in the sequence of Rap1A, we demonstrated that the tyrosine (Y) in position 32 is crucial in mediating the interaction of Rap1 N17 with the N-terminal of TRPM8. However, it was difficult to evaluate the functional effect of Y32 mutation in the TRPM8-mediated inhibition of Rap1 activation and cell adhesion, since this mutant seemed to have an intrinsic function on Rap1 activation, increasing it also in PC3 cells in the absence of TRPM8 (Figure 5e). These findings are in agreement with recent data that highlighted a central role of Y32 on GTP hydrolysis and effector binding of Ras protein, confirming the possibility that the residue Y32 not only plays a role in mediating Rap1-TRPM8 interaction but may also have an intrinsic functional role on Rap1 activation [89,90,91]. Indeed, Y32 falls into the so-called switch I, a loop region of small GTPase (residues 30–38) which, together with switch II (59–72), plays a pivotal role in the GTPase cycle. More in detail, it has been shown that substantial rearrangements of Switch I, Switch II, and the α3-helix occur during the GTPase switching from the inactive GDP-bound to the active GTP-bound state. These are attributable to a wide range of hydrogen-bonding networks which mainly lead to the loss of the functional water molecules, the positional shift of GTP, fluctuation of Y32, and translocation of Q61 [89]. Moreover, molecular dynamics simulations and Markov state models suggested that alteration of the Y32 chemical environment may cause notable changes in the conformation and dynamics of residues surrounding the active site [90]. Therefore, it is evident that Y32 dynamics and orientation can play a direct role in influencing GTPase’s properties, such as binding with GTP/GDP, the hydrolysis rate of GTP, and the affinity for regulators (GEF and GAP) as well as for effectors [90]. Consistently, c-Src was found to phosphorylate H/N/KRAS on conserved Tyr32, thus stalling their GTPase cycle in the GTP-bound state and affecting the binding to Raf effector as well as the downstream signaling [91,92]. Taking into account the highly conserved catalytic domain in the Ras-superfamily and the common conformational dynamics shared by Rap1 with Ras proteins [89], it seems possible to assume that tyrosine in position 32 can influence the GTP/GDP cycle of Rap1, influencing its activity as well as its interaction with modulators including TRPM8. Therefore, it is likely that the binding to TRPM8 could engage the hydroxyl group of Y32 and thus prevent the formation of the hydrogen bond with which Y32 contributes to the GTPase transition, and therefore, the adhesion process. 

Regarding the putative residues identified by molecular modeling in the TRPM8 sequence, i.e., glutamate (E) 207 and tyrosine (Y) 240, both the residues revealed a central role in the interaction with Rap1 N17 (Figure 6). Indeed, in the presence of TRPM8 mutants (TRPM8 E207A, TRPM8 Y240A, and TRPM8 E207A Y240A) TRPM8-Rap1 N17 interaction is significantly compromised, being definitively lost when TRPM8 E207A Y240A is combined to Rap1 N17 Y32A. Although a significant functional difference between TRPM8 wt and TRPM8 E207A Y240A on both cell adhesion and cell migration inhibition is evident, TRPM8 E207A Y240A still inhibits PC3 cells adhesion (Figure 7b,c). This observation could be explained by alternative and potentially Ca^2+^-mediated modalities disregarding the interaction with Rap1 by which TRPM8 exerts its inhibitory action on cell adhesion. This hypothesis is in agreement with the lack of inhibitory effect on cell adhesion of TRPM8 pore mutant in the presence of icilin (Figure 1e). Another possible explanation is that the two mutations on the TRPM8 sequence do not completely disrupt the interaction with endogenous Rap1A. The absence of a total reversion of the functional effects mediated by TRPM8 wt, in addition to the above reasons, could also be influenced by the efficiency of the transient transfection system used. In this regard, it is important to note that the cells were transfected with a rather low amount of TRPM8 plasmid in all experiments, to avoid system saturation with excessive channel expression, and consequently, to avoid the risk of masking the effects of TRPM8 double mutations in impairing TRPM8-Rap1A interaction. Finally, to further support our mechanistic model, we can affirm that the different functional behaviors shown by TRPM8 wt and double mutant, are not imputable to a different channel functionality since we verified that the two mutations in the cytosolic N-terminus did not affect the icilin-induced Ca^2+^ flux through the channel, neither qualitatively (Figure 7ai) nor quantitatively in terms of signal peak amplitude or percentage of responding cells (Figure 7aii). These data confirm once again the pore-independence of this pathway, showing that the inhibitory effect exerted by TRPM8 on Rap1 activation, and consequently on cell adhesion and migration, is, at least in part, due to physical interaction involving, in particular, the residues E207 and Y240 on the N-terminal cytosolic tail of the channel and the residue Y32 on the switch I region of the small GTPase.

To our best knowledge, there are no other examples in the literature of TRP-Rap1 interactions. Moreover, studies reporting TRP channels’ physical interaction with other small GTPases like Rac1 and RhoA [25,33,34] did not investigate the putative residues involved in such interactions except for TRPV5/6-Rab11a binding that has been localized in a conserved stretch in the TRPV carboxyl terminus [93]. Therefore, our work is one of the very few examples of a comprehensive characterization of the TRP-small GTPase interaction, revealing not only the biological impact of this interaction on cancer physiology but also which residues are determinant in this pathway and thus how potentially targeting it in cancer treatment.

Although the pore independence of these inhibitory mechanisms, icilin seems to have an active role in promoting TRPM8-Rap1 interaction, and consequently in the inhibition of Rap1 activation and PC3 cells’ migration/adhesion (Figure 1a–d, Figure 2b and Figure 7e). This rather surprising observation raises questions about the possible effects of agonists on TRPM8 in addition to pore gating and may be explained in light of the crystal structures (ligand-free and ligand-bound) of TRPM8 recently resolved by Yin and co-workers [48,94]. The authors reported cryo-electron microscopy structures (apo, icilin- and WS12-bound) of a full-length avian TRPM8 channel (TRPM8_FA_), with many features common to mammalian TRPM8 channels (83% sequence identity with human TRPM8), including cold- and menthol-sensitivity and pronounced dependence on phosphatidylinositol 4,5-bisphosphate (PIP_2_) [48]. Describing the structural basis for allosteric coupling between PIP_2_ and cooling agonists, they demonstrated that both icilin and PIP_2_ induce a conformational change by binding to a different region of segment 4b (S4b), that favors the bond of the other [94]. Therefore, the binding of PIP_2_ and icilin/WS12 triggers global conformational rearrangements in the TRPM8 transmembrane domain (TMD) that are propagated to the cytosolic domain (CD) via extensive intersubunit interactions observed between the TMD and the top layer of the CD ring, mainly mediated by the recently discovered “pre-S1 domain” specific of the TRPM8 structure [48,94]. Consequently, icilin is likely to favor and stabilize conformational changes in the TRPM8 N-terminal cytosolic tail where the binding to Rap1A occurs, due to the structural rearrangements associated with PIP_2_ binding, and thus may improve TRPM8-Rap1A physical and functional coupling.

Notably, our data suggested that the role of TRPM8 as a Rap1 inhibitor is not confined to ECs and PCa cells but may be generalized. Indeed, TRPM8-Rap1 interaction and function have been previously validated in ECs and also confirmed in an overexpression system of HEK cells [47]. Moreover, in this study, we demonstrated that the binding of TRPM8 with Rap1, mainly supported by E207 and Y240 residues in its N-terminal tail, affects cell adhesion not only in metastatic PCa cells but also in other cancer models like breast and cervical cancer, though to a different extent (Figure 8). The same is also true for the migratory behavior of breast cancer, but not for cervical cancer. Furthermore, neither the residues of TRPM8 involved in the interaction nor that of Rap1 are mutated in the cohort of patients of all types of cancer analyzed by us (Figure 4b). This bodes well for the potential use of TRPM8, or of the peptide sequence involved in the functional interaction with Rap1 alone, in cancer therapy to prevent and/or block its metastatic degeneration.

Nowadays, TRP channels interactome is an intriguing research field with great potential in cancer therapy. Indeed, disrupting or enhancing PPIs involving TRP channels could be a promising alternative therapeutic approach since allows targeting only the cellular pathways associated with a specific interaction thus minimizing side effects [95]. However, beyond the high specificity shown by peptides against their targets, this approach presents quite a few challenges. First, compared to targeting enzymes or receptors, targeting PPIs is complicated by the broad and less structured interface of these interactions [96]. Second, peptides have poor cell/tissue specificity and membrane penetration capacity. To overcome these limitations, in addition to a deeper mechanistic understanding of these interactions essential for improving the specificity and selectivity of this innovative approach; the combination of PPI-targeted bioactive peptides with cell-penetrating peptides (CPPs) successfully revealed good cellular uptake and biocompatibility with low side effects in vivo [97,98,99]. Interestingly, a mimetic of the Arg-Gly-Asp tripeptide epitope of fibrinogen able to bind to the αIIbβ3 integrin has been approved by FDA and is currently used in therapy as Tirofiban [100]. However, to date, no PPI modulators related to TRP channels have been approved for human treatment. Nevertheless, several in vivo studies have highlighted the great therapeutic potential of systemically-administered peptides capable of impairing the association between ion channel and their interactors [101,102,103,104]. In this context, a synthetic peptide based on PLC-γ1 SH2 domains, found to interact with TRPC1 [105], revealed an anti-tumoral activity in breast cancer [106]. Similarly, a peptide mimicking the γ-aminobutyric acid type A (GABAA) receptor-associated protein (GABARAP) which interacts with TRPV1 [107] has been proposed as a good candidate to promote TRPV1-associated suppression of breast cancer progression [108]. Furthermore, the design of peptides able to promote TRPV1-TRPA1 interaction could be a promising treatment for pain therapy [109]). Overall, growing evidence supports the use of peptidomimetics to improve current FDA-approved approaches to fighting cancer [110,111].

## 5. Conclusions

In this work, we clarified the molecular mechanism through which TRPM8 affects PCa progression by impairing cell motility. We demonstrated that in metastatic PCa cells TRPM8 acts as a sort of GDI-like protein, interacting with the inactive (GDP-bound) form of Rap1A and intracellularly sequestering it into the cytoplasm. This action hinders Rap1 translocation to the PM, where it normally is activated and exerts its positive action on cell adhesion, in principle by promoting the β_1_-integrin signaling pathway. Furthermore, we went deeper into the details of this interaction by identifying the residues of the two proteins mainly involved in its mediation: E207 and Y240 on the N-terminal cytosolic tail of the channel and the residue Y32 on the switch I region of the small GTPase. Validation of these residues on different cancer cell lines suggests a broad spectrum of action of this Ca^2+^-independent TRPM8-Rap1A interplay in terms of control of cell adhesion and migration. Altogether these findings provide new insights into potential therapeutic approaches involving TRPM8 as a target for cancer progression treatments.

## Figures and Tables

**Table 1:** Primers sequences used for single and double mutagenesis of TRPM8 (E207A and Y240A) and Rap1 (K31 and Y32A).

Vector	Mutation	Fw Primer	Rv Primer	Kit Used
pGEX-TRPM8 Nt	E207A	caatggccacaatattcgcctctgaactcctgctg	cagcaggagttcagaggcgaatattgtggccattg	Agilent kit
pGEX-TRPM8 Nt	Y240A	cttgtgaagtcatccataagggcctgggctaaaaaatagccctc	gagggctattttttagcccaggcccttatggatgacttcacaag	Agilent kit
pCDNA4-TRPM8	E207A	caatggccacaatattcgcctctgaactcctgctg	cagcaggagttcagaggcgaatattgtggccattg	Agilent kit
pCDNA4-TRPM8	Y240A	cttgtgaagtcatccataagggcctgggctaaaaaatagccctc	gagggctattttttagcccaggcccttatggatgacttcacaag	Agilent kit
pCMV-TNT-Rap1A	K31A	gaatcttctatcgttgggtcatatgcttcaacaaaaattccctgaacaaac	gtttgttcagggaatttttgttgaagcatatgacccaacgatagaagattc	Agilent kit
pCMV-TNT-Rap1A	Y32A	cttctatcgttgggtcagctttttcaacaaaaattccctgaacaaactgaac	gttcagtttgttcagggaatttttgttgaaaaagctgacccaacgatagaag	Agilent kit
pCMV-TNT-Rap1A	K31A	gaatcttctatcgttgggtcatatgcttcaacaaaaattccctgaacaaac	gtttgttcagggaatttttgttgaagcatatgacccaacgatagaagattc	Agilent kit
pCMV-TNT-Rap1A	Y32A	cttctatcgttgggtcagctttttcaacaaaaattccctgaacaaactgaac	gttcagtttgttcagggaatttttgttgaaaaagctgacccaacgatagaag	Agilent kit
peGFP-Rap1A-N17	K31A	agttcagaggcgaatattgtgg	cctgctgatggtgttatc	NEB
peGFP-Rap1A-N17	Y32A	tttagcccaggcccttatggatgac	aaatagccctcagcatcg	NEB
pMT2SM-HA-Rap1A	K31A	gaatcttctatcgttgggtcatatgcttcaacaaaaattccctgaacaaac	gtttgttcagggaatttttgttgaagcatatgacccaacgatagaagattc	Agilent kit
pMT2SM-HA-Rap1A	Y32A	cttctatcgttgggtcagctttttcaacaaaaattccctgaacaaactgaac	gttcagtttgttcagggaatttttgttgaaaaagctgacccaacgatagaag	Agilent kit
pMT2SM-HA-Rap1A N17	K31A	gaatcttctatcgttgggtcatatgcttcaacaaaaattccctgaacaaac	gtttgttcagggaatttttgttgaagcatatgacccaacgatagaagattc	Agilent kit
pMT2SM-HA-Rap1A N17	Y32A	cttctatcgttgggtcagctttttcaacaaaaattccctgaacaaactgaac	gttcagtttgttcagggaatttttgttgaaaaagctgacccaacgatagaag	Agilent kit

## Data Availability

The data presented in this study are available in this article (and supplementary material).

## Supplementary Materials

The following are available online at https://www.mdpi.com/article/10.3390/cancers14092261/s1, Figure S1: Validation of TRPM8 pore mutant; Figure S2: Original uncropped blot referring to Figure 2ai; Figure S3: TRPM8 and Rap1A basal expression in PC3, MCF-7, and HeLa cell lines; Figure S4: RCA of the interaction between TRPM8 and Rap1A; Figure S5: Role of K31 and Y32 in mediating the direct interaction between the inactive (GDP-bound) form of Rap1 and TRPM8 N terminus; Figure S6: Original uncropped blots referring to Figure 6 and Figure 7; Figure S7: TRPM8-Rap1A interaction in cancer cells viability; Figure S8: TRPM8-Rap1A interaction in healthy and cancerous prostate tissues.

## References

[1] Sung H., Ferlay J., Siegel R.L., Laversanne M., Soerjomataram I., Jemal A., Bray F. (2021). Global Cancer Statistics 2020: GLOBOCAN Estimates of Incidence and Mortality Worldwide for 36 Cancers in 185 Countries. CA. Cancer J. Clin..

[2] Gardel M.L., Schneider I.C., Aratyn-Schaus Y., Waterman C.M. (2010). Mechanical integration of actin and adhesion dynamics in cell migration. Annu. Rev. Cell Dev. Biol..

[3] Parsons J.T., Horwitz A.R., Schwartz M.A. (2010). Cell adhesion: Integrating cytoskeletal dynamics and cellular tension. Nat. Rev. Mol. Cell Biol..

[4] Gkika D., Prevarskaya N. (2011). TRP channels in prostate cancer: The good, the bad and the ugly?. Asian J. Androl..

[5] Fiorio Pla A., Gkika D. (2013). Emerging role of TRP channels in cell migration: From tumor vascularization to metastasis. Front. Physiol..

[6] Bernardini M., Fiorio Pla A., Prevarskaya N., Gkika D. (2015). Human transient receptor potential (TRP) channels expression profiling in carcinogenesis. Int. J. Dev. Biol..

[7] Lastraioli E., Iorio J., Arcangeli A. (2015). Ion channel expression as promising cancer biomarker. Biochim. Biophys. Acta—Biomembr..

[8] Litan A., Langhans S.A. (2015). Cancer as a channelopathy: Ion channels and pumps in tumor development and progression. Front. Cell. Neurosci..

[9] Chinigò G., Castel H., Chever O., Gkika D. (2021). TRP Channels in Brain Tumors. Front. Cell Dev. Biol..

[10] Tsavaler L., Shapero M.H., Morkowski S., Laus R. (2001). Trp-p8, a novel prostate-specific gene, is up-regulated in prostate cancer and other malignancies and shares high homology with transient receptor potential calcium channel proteins. Cancer Res..

[11] Shapovalov G., Ritaine A., Skryma R., Prevarskaya N. (2016). Role of TRP ion channels in cancer and tumorigenesis. Semin. Immunopathol..

[12] Iamshanova O., Fiorio Pla A., Prevarskaya N. (2017). Molecular mechanisms of tumour invasion: Regulation by calcium signals. J. Physiol..

[13] Vrenken K.S., Jalink K., van Leeuwen F.N., Middelbeek J. (2015). Beyond ion-conduction: Channel-dependent and -independent roles of TRP channels during development and tissue homeostasis. Biochim. Biophys. Acta—Mol. Cell Res..

[14] Bödding M. (2007). TRP proteins and cancer. Cell. Signal..

[15] Van De Graaf S.F.J., Hoenderop J.G.J., Bindels R.J.M. (2006). Regulation of TRPV5 and TRPV6 by associated proteins. Am. J. Physiol. Renal Physiol..

[16] Gkika D., Mahieu F., Niliur B., Hoenderop J.G.J., Bindels R.J.M. (2004). 80K-H as a new Ca^2+^ sensor regulating the activity of the epithelial Ca^2+^ channel transient receptor potential cation channel V5 (TRPV5). J. Biol. Chem..

[17] Gkika D., Topala C.N., Hoenderop J.G.J., Bindels R.J.M. (2006). The immunophilin FKBP52 inhibits the activity of the epithelial Ca^2+^ channel TRPV5. Am. J. Physiol. Renal Physiol..

[18] Gkika D., Topala C.N., Chang Q., Picard N., Thébault S., Houillier P., Hoenderop J.G.J., Bindels R.J.M. (2006). Tissue kallikrein stimulates Ca^2+^ reabsorption via PKC-dependent plasma membrane accumulation of TRPV5. EMBO J..

[19] Sinkins W.G., Goel M., Estacion M., Schilling W.P. (2004). Association of immunophilins with mammalian TRPC channels. J. Biol. Chem..

[20] Chang Q., Hoefs S., Van Der Kemp A.W., Topala C.N., Bindels R.J., Hoenderop J.G. (2005). The beta-glucuronidase klotho hydrolyzes and activates the TRPV5 channel. Science.

[21] Chinigò G., Fiorio Pla A., Gkika D. (2020). TRP Channels and Small GTPases Interplay in the Main Hallmarks of Metastatic Cancer. Front. Pharmacol..

[22] Guéguinou M., Harnois T., Crottes D., Uguen A., Deliot N., Gambade A., Chantôme A., Haelters J.P., Jaffrès P.A., Jourdan M.L. (2016). SK3/TRPC1/Orai1 complex regulates SOCE-dependent colon cancer cell migration: A novel opportunity to modulate anti-EGFR mAb action by the alkyl-lipid Ohmline. Oncotarget.

[23] Bezzerides V.J., Ramsey I.S., Kotecha S., Greka A., Clapham D.E. (2004). Rapid vesicular translocation and insertion of TRP channels. Nat. Cell Biol..

[24] Singh I., Knezevic N., Ahmmed G.U., Kini V., Malik A.B., Mehta D. (2007). Gαq-TRPC6-mediated Ca 2+ Entry Induces RhoA Activation and Resultant Endothelial Cell Shape Change in Response to Thrombin. J. Biol. Chem..

[25] Tian D., Jacobo S.M.P., Billing D., Rozkalne A., Gage S.D., Anagnostou T., Pavenstädt H., Hsu H., Schlondorff J., Ramos A. (2010). Antagonistic regulation od actin dynamics and cell motility by TRPC5 and TRPC6 channels. Sci. Signal..

[26] Su L.T., Liu W., Chen H.C., González-Pagán O., Habas R., Runnels L.W. (2011). TRPM7 regulates polarized cell movements. Biochem. J..

[27] Tomasek J.J., Vaughan M.B., Kropp B.P., Gabbiani G., Martin M.D., Haaksma C.J., Hinz B. (2006). Contraction of myofibroblasts in granulation tissue is dependent on Rho/Rho kinase/myosin light chain phosphatase activity. Wound Repair. Regen..

[28] Adapala R.K., Thoppil R.J., Luther D.J., Paruchuri S., Meszaros J.G., Chilian W.M., Thodeti C.K. (2013). Cellular Cardiology TRPV4 channels mediate cardiac fi broblast differentiation by integrating mechanical and soluble signals. J. Mol. Cell. Cardiol..

[29] Lee W.H., Choong L.Y., Mon N.N., Lu S., Lin Q., Pang B., Yan B., Sri V., Krishna R., Singh H. (2016). TRPV4 regulates breast cancer cell extravasation, stiffness and actin cortex. Sci. Rep..

[30] Ou-yang Q., Li B., Xu M., Liang H. (2018). TRPV4 promotes the migration and invasion of glioma cells via AKT/Rac1 signaling. Biochem. Biophys. Res. Commun..

[31] Gao G., Wang W., Tadagavadi R.K., Briley N.E., Love M.I., Miller B.A., Reeves W.B. (2014). TRPM2 mediates ischemic kidney injury and oxidant stress through RAC1. J. Clin. Investig..

[32] Laragione T., Harris C., Gulko P.S. (2019). TRPV2 suppresses Rac1 and RhoA activation and invasion in rheumatoid arthritis fibroblast-like synoviocytes. Int. Immunopharmacol..

[33] Mehta D., Ahmmed G.U., Paria B.C., Holinstat M., Voyno-Yasenetskaya T., Tiruppathi C., Minshall R.D., Malik A.B. (2003). RhoA interaction with inositol 1,4,5-trisphosphate receptor and transient receptor potential channel-1 regulates Ca^2+^ entry: Role in signaling increased endothelial permeability. J. Biol. Chem..

[34] Chung H.K., Rathor N., Wang S.R., Wang J.Y., Rao J.N. (2015). RhoA enhances store-operated Ca^2+^ entry and intestinal epithelial restitution by interacting with TRPC1 after wounding. Am. J. Physiol.—Gastrointest. Liver Physiol..

[35] Nagasawa M., Kojima I. (2015). Translocation of TRPV2 channel induced by focal administration of mechanical stress. Physiol. Rep..

[36] Sun J., Yang T., Wang P., Ma S., Zhu Z., Pu Y., Li L., Zhao Y., Xiong S., Liu D. (2014). Activation of cold-sensing transient receptor potential melastatin subtype 8 antagonizes vasoconstriction and hypertension through attenuating RhoA/Rho kinase pathway. Hypertension.

[37] Thoppil R.J., Cappelli H.C., Adapala R.K., Kanugula A.K., Paruchuri S., Thodeti C.K. (2016). TRPV4 channels regulate tumor angiogenesis via modulation of Rho/Rho kinase pathway. Oncotarget.

[38] Feldman B.J., Feldman D. (2001). The development of androgen-independent prostate cancer. Nat. Rev. Cancer.

[39] Henshall S.M., Afar D.E.H., Hiller J., Horvath L.G., Quinn D.I., Rasiah K.K., Gish K., Willhite D., Kench J.G., Gardiner-Garden M. (2003). Survival analysis of genome-wide gene expression profiles of prostate cancers identifies new prognostic targets of disease relapse. Cancer Res..

[40] Grolez G.P., Gordiendko D.V., Clarisse M., Hammadi M., Desruelles E., Fromont G., Prevarskaya N., Slomianny C., Gkika D. (2019). TRPM8-androgen receptor association within lipid rafts promotes prostate cancer cell migration. Cell Death Dis..

[41] Bidaux G., Roudbaraki M., Merle C., Crépin A., Delcourt P., Slomianny C., Thebault S., Bonnal J.L., Benahmed M., Cabon F. (2005). Evidence for specific TRPM8 expression in human prostate secretory epithelial cells: Functional androgen receptor requirement. Endocr. Relat. Cancer.

[42] Zhang L., Barritt G.J. (2004). Evidence that TRPM8 is an androgen-dependent Ca^2+^ channel required for the survival of prostate cancer cells. Cancer Res..

[43] Gkika D., Flourakis M., Lemonnier L., Prevarskaya N. (2010). PSA reduces prostate cancer cell motility by stimulating TRPM8 activity and plasma membrane expression. Oncogene.

[44] Gkika D., Lemonnier L., Shapovalov G., Gordienko D., Poux C., Bernardini M., Bokhobza A., Bidaux G., Degerny C., Verreman K. (2015). TRP channel-associated factors are a novel protein family that regulates TRPM8 trafficking and activity. J. Cell Biol..

[45] Grolez G.P., Hammadi M., Barras A., Gordienko D., Slomianny C., Völkel P., Angrand P.O., Pinault M., Guimaraes C., Potier-Cartereau M. (2019). Encapsulation of a TRPM8 Agonist, WS12, in Lipid Nanocapsules Potentiates PC3 Prostate Cancer Cell Migration Inhibition through Channel Activation. Sci. Rep..

[46] Yang Z.H., Wang X.H., Wang H.P., Hu L.Q. (2009). Effects of TRPM8 on the proliferation and motility of prostate cancer PC-3 cells. Asian J. Androl..

[47] Genova T., Grolez G.P.G.P., Camillo C., Bernardini M., Bokhobza A., Richard E., Scianna M., Lemonnier L., Valdembri D., Munaron L. (2017). TRPM8 inhibits endothelial cell migration via a nonchannel function by trapping the small GTPase Rap1. J. Cell Biol..

[48] Yin Y., Wu M., Zubcevic L., Borschel W.F., Lander G.C., Lee S.Y. (2018). Structure of the cold- and menthol-sensing ion channel TRPM8. Science.

[49] Paulsen C., Armache J., Gao Y., Cheng Y., Julius D. (2015). Structure of the TRPA1 ion channel suggests regulatory mechanisms. Nature.

[50] Sali A., Blundell T. (1993). Comparative protein modelling by satisfaction of spatial restraints. J. Mol. Biol..

[51] Nassar N., Horn G., Herrmann C.A., Scherer A., McCormick F., Wittinghofer A. (1995). The 2.2 Å crystal structure of the Ras-binding domain of the serine/threonine kinase c-Raf1 in complex with RaplA and a GTP analogue. Nature.

[52] Kozakov D., Hall D., Xia B., Porter K., Padhorny D., Yueh C., Beglov D., Vajda S. (2017). The ClusPro web server for protein-protein docking. Nat. Protoc..

[53] Quignot C., Rey J., Yu J., Tufféry P., Guerois R., Andreani J. (2018). InterEvDock2: An expanded server for protein docking using evolutionary and biological information from homology models and multimeric inputs. Nucleic Acids Res..

[54] Brysbaert G., Lorgouilloux K., Vranken W., Lensink M. (2018). RINspector: A Cytoscape app for centrality analyses and DynaMine flexibility prediction. Bioinformatics.

[55] Brysbaert G., Mauri T., de Ruyck J., Lensink M. (2019). Identification of Key Residues in Proteins Through Centrality Analysis and Flexibility Prediction with RINspector. Curr. Protoc. Bioinforma..

[56] Shannon P., Markiel A., Ozier O., Baliga N., Wang J., Ramage D., Amin N., Schwikowski B., Ideker T. (2003). Cytoscape: A software environment for integrated models of biomolecular interaction networks. Genome Res..

[57] Su G., Morris J., Demchak B., Bader G. (2014). Biological network exploration with Cytoscape 3. Curr. Protoc. Bioinforma..

[58] Amitai G., Shemesh A., Sitbon E., Shklar M., Netanely D., Venger I., Pietrokovski S. (2004). Network analysis of protein structures identifies functional residues. J. Mol. Biol..

[59] Del Sol A., Fujihashi H., Amoros D., Nussinov R. (2006). Residues crucial for maintaining short paths in network communication mediate signaling in proteins. Mol. Syst. Biol..

[60] Bivona T.G., Wiener H.H., Ahearn I.M., Silletti J., Chiu V.K., Philips M.R. (2004). Rap1 up-regulation and activation on plasma membrane regulates T cell adhesion. J. Cell Biol..

[61] Bivona T.G., Quatela S., Philips M.R. (2006). Analysis of Ras Activation in Living Cells with GFP-RBD. Methods Enzymol..

[62] Bernardini M., Brossa A., Chinigo G., Grolez G.P., Trimaglio G., Allart L., Hulot A., Marot G., Genova T., Joshi A. (2019). Signatures in Tumor-Derived Endothelial Cells: Functional Roles in Prostate Cancer Angiogenesis. Cancers.

[63] Suzuki J., Kanemaru K., Ishii K., Ohkura M., Okubo Y., Iino M. (2014). Imaging intraorganellar Ca^2+^ at subcellular resolution using CEPIA. Nat. Commun..

[64] https://xenabrowser.net/datapages/?dataset=GDC-%0APANCAN.mutect2_snv.tsv&host=https%3A%2F%2Fgdc.xenahubs.net&removeHub=https%0A%3A%2F%2Fxena.treehouse.gi.ucsc.edu%3A443.

[65] Edmund. https://github.com/MrHedmad/Edmund.

[66] https://www.ensembl.org/Homo_sapiens/Gene/Summary?db=core;g=ENSG000001164%0A73;r=1:111542218-111716691.

[67] https://www.ensembl.org/Homo_sapiens/Gene/Summary?db=core;g=ENSG000001444%0A81;r=2:233917373-234019522.

[68] ANOVA_merged.R. https://gist.github.com/MrHedmad/a53eb36ff1233e45bc638008912c0d35.

[69] Bidaux G., Flourakis M., Thebault S., Zholos A., Beck B., Gkika D., Roudbaraki M., Bonnal J.L., Mauroy B., Shuba Y. (2007). Prostate cell differentiation status determines transient receptor potential melastatin member 8 channel subcellular localization and function. J. Clin. Investig..

[70] Pillozzi S., Brizzi M.F., Bernabei P.A., Bartolozzi B., Caporale R., Basile V., Boddi V., Pegoraro L., Becchetti A., Arcangeli A. (2007). VEGFR-1 (FLT-1), β1 integrin, and hERG K+ channel for a macromolecular signaling complex in acute myeloid leukemia: Role in cell migration and clinical outcome. Blood.

[71] Negri S., Faris P., Maniezzi C., Pellavio G., Spaiardi P., Botta L., Laforenza U., Biella G., Moccia D.F. (2021). NMDA receptors elicit flux-independent intracellular Ca^2+^ signals via metabotropic glutamate receptors and flux-dependent nitric oxide release in human brain microvascular endothelial cells. Cell Calcium.

[72] Desai B.N., Krapivinsky G., Navarro B., Krapivinsky L., Carter B.C., Febvay S., Delling M., Penumaka A., Ramsey I.S., Manasian Y. (2012). Cleavage of TRPM7 Releases the Kinase Domain from the Ion Channel and Regulates Its Participation in Fas-Induced Apoptosis. Dev. Cell.

[73] Faouzi M., Kilch T., Horgen F.D., Fleig A., Penner R. (2017). The TRPM7 channel kinase regulates store-operated calcium entry. J. Physiol..

[74] Joly D., Ishibe S., Nickel C., Yu Z., Somlo S., Cantley L. (2006). The polycystin 1-C-terminal fragment stimulates ERK-dependent spreading of renal epithelial cells. J. Biol. Chem..

[75] Bos J.L. (2005). Linking Rap to cell adhesion. Curr. Opin. Cell Biol..

[76] Boettner B., Van Aelst L. (2009). Control of cell adhesion dynamics by Rap1 signaling. Curr. Opin. Cell Biol..

[77] Moissoglu K., Schwartz M.A. (2014). Spatial and temporal control of Rho GTPase functions. Cell. Logist..

[78] Yarwood S., Cullen P.J., Kupzig S. (2006). The GAP1 family of GTPase-activating proteins: Spatial and temporal regulators of small GTPase signalling. Biochem. Soc. Trans..

[79] Cherfils J., Zeghouf M. (2013). Regulation of small GTPases by GEFs, GAPs, and GDIs. Physiol. Rev..

[80] Reedquist K.A., Ross E., Koop E.A., Wolthuis R.M.F., Zwartkruis F.J.T., Van Kooyk Y., Salmon M., Buckley C.D., Bos J.L. (2000). The small GTPase, Rap1, mediates CD31-induced integrin adhesion. J. Cell Biol..

[81] Carmona G., Go S., Orlandi A., Ba T., Jugold M., Kiessling F., Henschler R., Zeiher A.M., Dimmeler S., Chavakis E. (2009). Role of the small GTPase Rap1 for integrin activity regulation in endothelial cells and angiogenesis. Blood J. Am. Soc. Hematol..

[82] Prevarskaya N., Zhang L., Barritt G. (2007). TRP channels in cancer. Biochim. Biophys. Acta—Mol. Basis Dis..

[83] Skryma R., Mariot P., Bourhis X., Coppenolle F., Shuba Y., Abeele F.V., Legrand G., Humez S., Boilly B., Prevarskaya N. (2000). Store depletion and store-operated Ca^2+^ current in human prostate cancer LNCaP cells: Involvement in apoptosis. J. Physiol..

[84] Thebault S., Lemonnier L., Bidaux G., Flourakis M., Bavencoffe A., Gordienko D., Roudbaraki M., Delcourt P., Panchin Y., Shuba Y. (2005). Novel role of cold/menthol-sensitive transient receptor potential melastatine family member 8 (TRPM8) in the activation of store-operated channels in LNCaP human prostate cancer epithelial cells. J. Biol. Chem..

[85] Abeele F.V., Skryma R., Shuba Y., Van Coppenolle F., Slomianny C., Roudbaraki M., Mauroy B., Wuytack F., Prevarskaya N. (2002). Bcl-2-dependent modulation of Ca^2+^ homeostasis and store-operated channels in prostate cancer cells. Cancer Cell.

[86] Vanoverberghe K., Abeele F.V., Mariot P., Lepage G., Roudbaraki M., Bonnal J.L., Mauroy B., Shuba Y., Skryma R., Prevarskaya N. (2004). Ca^2+^ homeostasis and apoptotic resistance of neuroendocrine-differentiated prostate cancer cells. Cell Death Differ..

[87] Zhang X., Mak S., Li L., Parra A., Denlinger B., Belmonte C., McNaughton P.A. (2012). Direct inhibition of the cold-Activated TRPM8 ion channel by Gα q. Nat. Cell Biol..

[88] Klasen K., Hollatz D., Zielke S., Gisselmann G., Hatt H., Wetzel C.H. (2012). The TRPM8 ion channel comprises direct Gq protein-activating capacity. Eur. J. Physiol..

[89] Matsumoto S., Miyano N., Baba S., Liao J., Kawamura T., Tsuda C., Takeda A., Yamamoto M., Kumasaka T., Kataoka T. (2016). Molecular Mechanism for Conformational Dynamics of Ras·GTP Elucidated from In-Situ Structural Transition in Crystal. Sci. Rep..

[90] Khaled M., Gorfe A., Sayyed-Ahmad A. (2019). Conformational and Dynamical Effects of Tyr32 Phosphorylation in K-Ras: Molecular Dynamics Simulation and Markov State Models Analysis. J. Phys. Chem. B.

[91] Kano Y., Gebregiworgis T., Marshall C.B., Radulovich N., Poon B.P.K., St-Germain J., Cook J.D., Valencia-Sama I., Grant B.M.M., Herrera S.G. (2019). Tyrosyl phosphorylation of KRAS stalls GTPase cycle via alteration of switch I and II conformation. Nat. Commun..

[92] Bunda S., Heir P., Srikumar T., Cook J.D., Burrell K., Kano Y., Lee J.E., Zadeh G., Raught B., Ohh M. (2014). Src promotes GTPase activity of Ras via tyrosine 32 phosphorylation. Proc. Natl. Acad. Sci. USA.

[93] Van de Graaf S.F.J., Chang Q., Mensenkamp A.R., Hoenderop J.G.J., Bindels R.J.M. (2006). Direct Interaction with Rab11a Targets the Epithelial Ca 2+ Channels TRPV5 and TRPV6 to the Plasma Membrane. Mol. Cell. Biol..

[94] Yin Y., Le S.C., Hsu A.L., Borgnia M.J., Yang H., Lee S.Y. (2019). Structural basis of cooling agent and lipid sensing by the cold-activated TRPM8 channel. Science.

[95] Mabonga L., Kappo A.P. (2019). Protein-protein interaction modulators: Advances, successes and remaining challenges. Biophys. Rev..

[96] Scott D.E., Bayly A.R., Abell C., Skidmore J. (2016). Small molecules, big targets: Drug discovery faces the protein-protein interaction challenge. Nat. Rev. Drug Discov..

[97] Habault J., Poyet J.L. (2019). Recent advances in cell penetrating peptide-based anticancer therapies. Molecules.

[98] Xie J., Bi Y., Zhang H., Dong S., Teng L., Lee R.J., Yang Z. (2020). Cell-Penetrating Peptides in Diagnosis and Treatment of Human Diseases: From Preclinical Research to Clinical Application. Front. Pharmacol..

[99] Kang R.H., Jang J.E., Huh E., Kang S.J., Ahn D.R., Kang J.S., Sailor M.J., Yeo S.G., Oh M.S., Kim D. (2020). A brain tumor-homing tetra-peptide delivers a nano-therapeutic for more effective treatment of a mouse model of glioblastoma. Nanoscale Horizons.

[100] Bledzka K., Smyth S.S., Plow E.F. (2013). Integrin alphaIIbbeta3: From discovery to efficacious therapeutic target. Circ. Res..

[101] Fisher M., Btesh J., McNaughton P. (2013). Disrupting Sensitization of Transient Receptor Potential Vanilloid Subtype 1 Inhibits Inflammatory Hyperalgesia. J. Neurosci..

[102] Schulie A.J., Yeh C.Y., Orang B.N., Pa O.J., Hopkin M.P., Moutal A., Khanna R., Sun D., Justic J.A., Aizenman E. (2020). Targeted disruption of Kv2.1-VAPA association provides neuroprotection against ischemic stroke in mice by declustering Kv2.1 channels. Sci. Adv..

[103] Brittain J.M., Duarte D.B., Wilson S.M., Zhu W., Ballard C., Johnson P.L., Liu N., Xiong W., Ripsch M.S., Wang Y. (2011). Suppression of inflammatory and neuropathic pain by uncoupling CRMP-2 from the presynaptic Ca^2+^ channel complex. Nat. Med..

[104] Fischer A., Rosen A.C., Ensslin C.J., Wu S., Lacouture M.E. (2013). Pruritus to anticancer agents targeting the EGFR, BRAF, and CTLA-4. Dermatol. Ther..

[105] Tu C.L., Chang W., Bikle D.D. (2005). Phospholipase Cγ1 is required for activation of store-operated channels in human keratinocytes. J. Investig. Dermatol..

[106] Katterle Y., Brandt B.H., Dowdy S.F., Niggemann B., Zänker K.S., Dittmar T. (2004). Antitumour effects of PLC-γ1-(SH2)2-TAT fusion proteins on EGFR/c-erbB-2-positive breast cancer cells. Br. J. Cancer.

[107] Laínez S., Valente P., Ontoria-Oviedo I., Estévez-Herrera J., Camprubí-Robles M., Ferrer-Montiel A., Planells-Cases R. (2010). GABA A receptor associated protein (GABARAP) modulates TRPV1 expression and channel function and desensitization. FASEB J..

[108] Saldías M.P., Maureira D., Orellana-Serradell O., Silva I., Lavanderos B., Cruz P., Torres C., Cáceres M., Cerda O. (2021). TRP Channels Interactome as a Novel Therapeutic Target in Breast Cancer. Front. Oncol..

[109] Weng H.J., Patel K.N., Jeske N.A., Bierbower S.M., Zou W., Tiwari V., Zheng Q., Tang Z., Mo G.C.H., Wang Y. (2015). Tmem100 Is a Regulator of TRPA1-TRPV1 Complex and Contributes to Persistent Pain. Neuron.

[110] Mabonga L., Kappo A.P. (2020). Peptidomimetics: A Synthetic Tool for Inhibiting Protein–Protein Interactions in Cancer. Int. J. Pept. Res. Ther..

[111] Corbi-Verge C., Kim P.M. (2016). Motif mediated protein-protein interactions as drug targets. Cell Commun. Signal..

