# Supplementary material for: TRPM8-Rap1A Interaction Sites as Critical Determinants for Adhesion and Migration of Prostate and Other Epithelial Cancer Cells
Source: Cancers (Basel). 2022 Apr 30;14(9):2261. doi: 10.3390/cancers14092261 (PMC9102551; doi:10.3390/cancers14092261)
Supplement: Supplementary file 1 [file cancers-14-02261-s001.zip › cancers-1657936-supplementary.pdf]

Supplementary materials

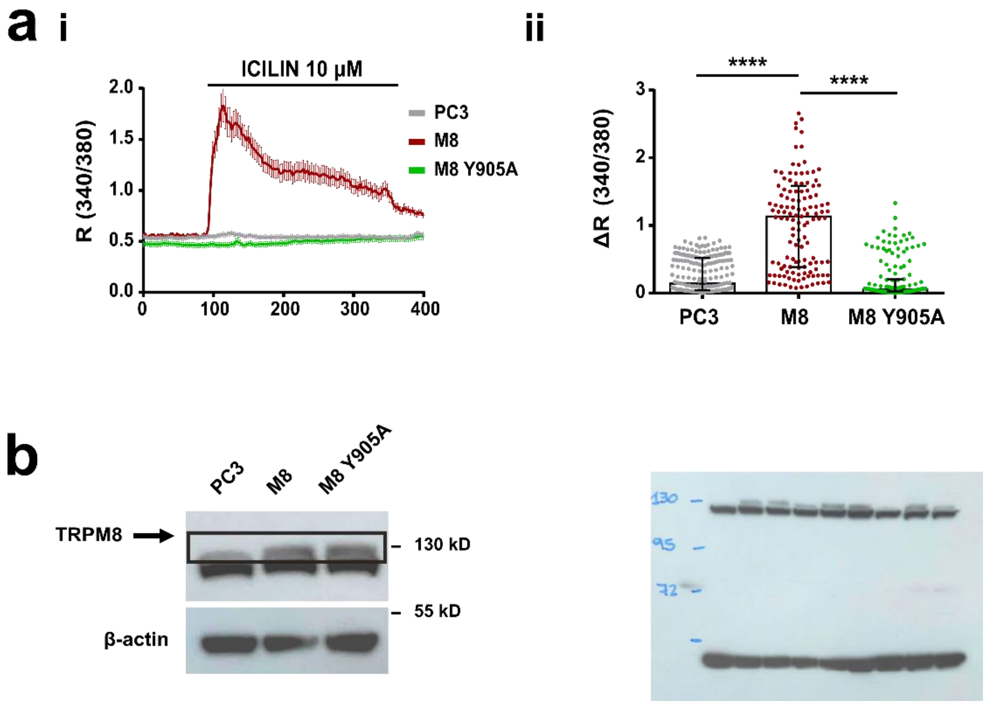

Figure S1. Validation of TRPM8 pore mutant.

(**a i**) Ca^{2+}-imaging traces in response to TRPM8 agonist (10 µM icilin) in PC3 (grey), PC3 transiently overexpressing TRPM8 (red) and PC3 transiently overexpressing the pore mutant TRPM8 Y905A (green). Traces represent mean ± SEM of cells in the recorded field of one representative experiment (n=33 for PC3, n=24 for M8 and n=25 for M8 Y905A). (**ii**) Scatter dot plot showing peak amplitude of icilin-mediated Ca^{2+} responses (median with interquartile range of different cells in the field from at least 5 independent experiments). All cells were considered for PC3 and PC3 overexpressing TRPM8 Y905A analysis, only icilin-responsive cells were considered for PC3 overexpressing TRPM8 (n=225 for PC3, n=135 for M8 and n=159 for M8 Y905A). Statistical significance versus PC3 or PC3 overexpressing TRPM8, ****: P < 0.0001 (Kruskal-Wallis test with *post-hoc* Dunn's test).
(**b**) Representative immunoblot showing TRPM8 expression (rabbit anti-TRPM8 Ab109308 1:800) in PC3 cells, PC3 cells overexpressing TRPM8 wt (0.625 µg) and PC3 cells overexpressing TRPM8 Y905A (0.625 µg). β-actin (mouse anti-β-actin Sigma-Aldrich A5316 1:1000) was used as a loading control; the specific bands referring to TRPM8 expression are framed and indicated by the arrows.
The full uncropped blot is reported on the right. Samples order (from left to right): 1) PC3 CNTRL; 2) PC3+TRPM8 wt; 3) PC3+TRPM8 Y905A; 4) MCF-7 CNTRL; 5) MCF-7+TRPM8 wt; 6) MCF-7+TRPM8 E207A Y240A; 7) HeLa CNTRL; 8) HeLa+TRPM8 wt; 9) HeLa+TRPM8 E207A Y240A

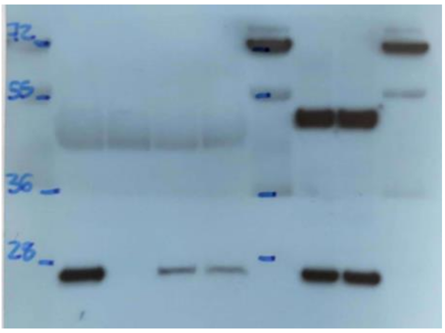

Figure S2. Original uncropped blot referring to Fig. 2ai.

Samples order (from left to right): 1) GTP Ɣs: 2) GDP; 3) PC3 CNTRL (pulled-down PD); 4) TRPM8 (PD); 5) empty; 6) PC3 CNTRL (total lysates TL); 7) TRPM8 (TL)

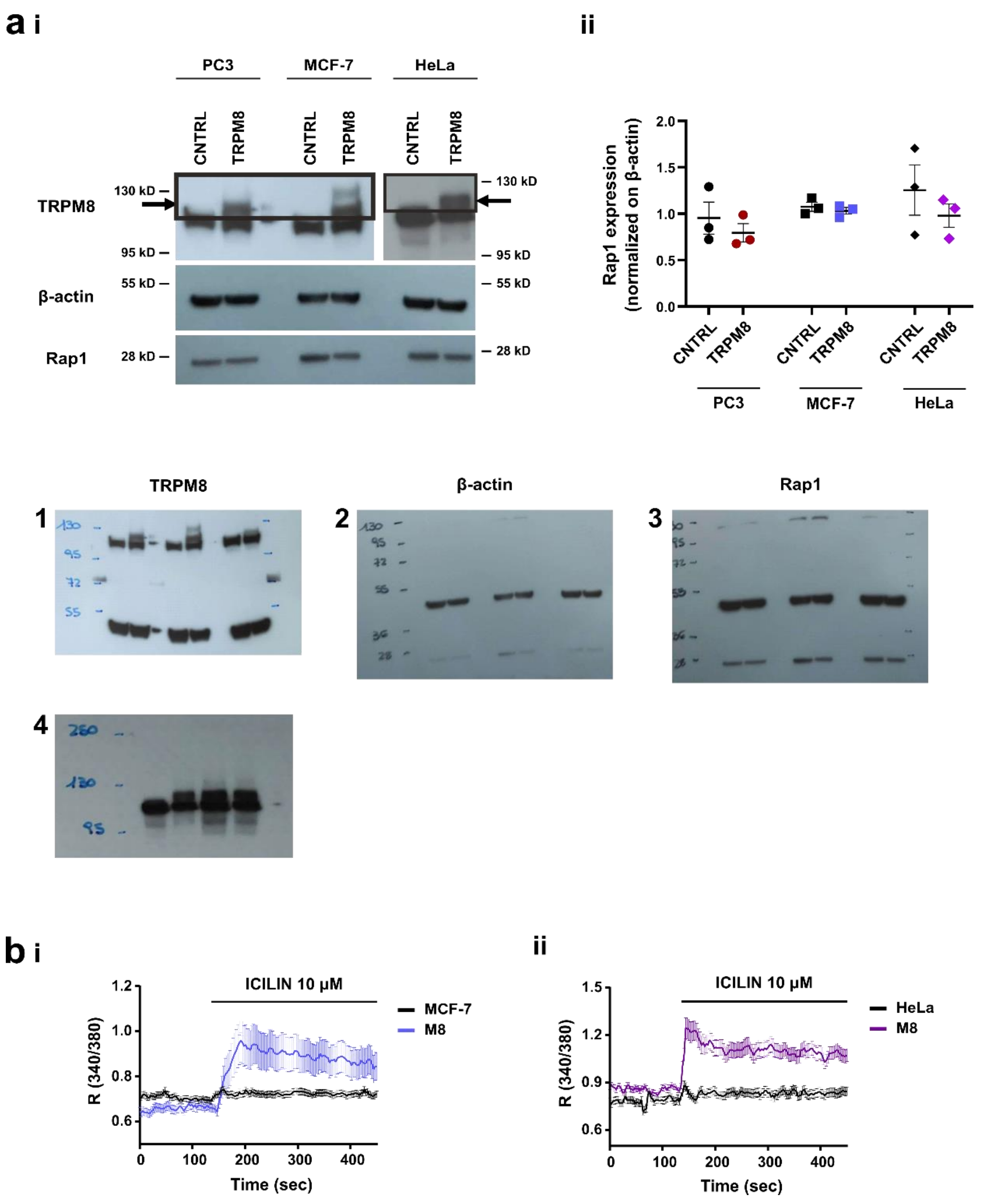

Figure S3. TRPM8 and Rap1A basal expression in PC3, MCF-7, and HeLa cell lines.

(**a i**) Representative immunoblot showing TRPM8 (rabbit anti-TRPM8 Ab109308 1:800) and Rap1 (rabbit anti-Rap1 Thermo Fisher scientific 1862344) expression in PC3, MCF-7, and HeLa cells wt and overexpressing exogenous TRPM8 wt (0.625 μg). The specific bands referring to TRPM8 expression are framed and indicated by the arrows. β-actin (mouse anti-β-actin Sigma-Aldrich A5316 1:1000) was used as a loading control and to normalize and quantify (**ii**) the endogenous amount of Rap1 within cells overexpressing or not TRPM8 (n=3). The original uncropped blots are reported below; samples order in blots 1-3 (from left to right): 1) PC3 wt; 2) PC3+TRPM8; 3) empty; 4) MCF-7 wt; 5) MCF-7+TRPM8; 6) empty; 7) HeLa wt; 8) HeLa+TRPM8; samples order in blot 4 (from left to right): 1) HeLa wt; 2) Hela+TRPM8 (10 μg); 3) HeLa+TRPM8 (30 μg); 4) 3) HeLa+TRPM8 (20 μg).
(**b**) Ca^{2+}-imaging traces in response to TRPM8 agonist (10 μM icilin) in MCF-7 (**i**) and HeLa (**ii**), transiently overexpressing or not TRPM8. Traces represent mean ± SEM of cells in the recorded field of one representative experiment (n=38 for MCF-7 wt, n=29 for MCF-7 M8; n=22 for HeLa CNTRL, n=29 for HeLa M8).

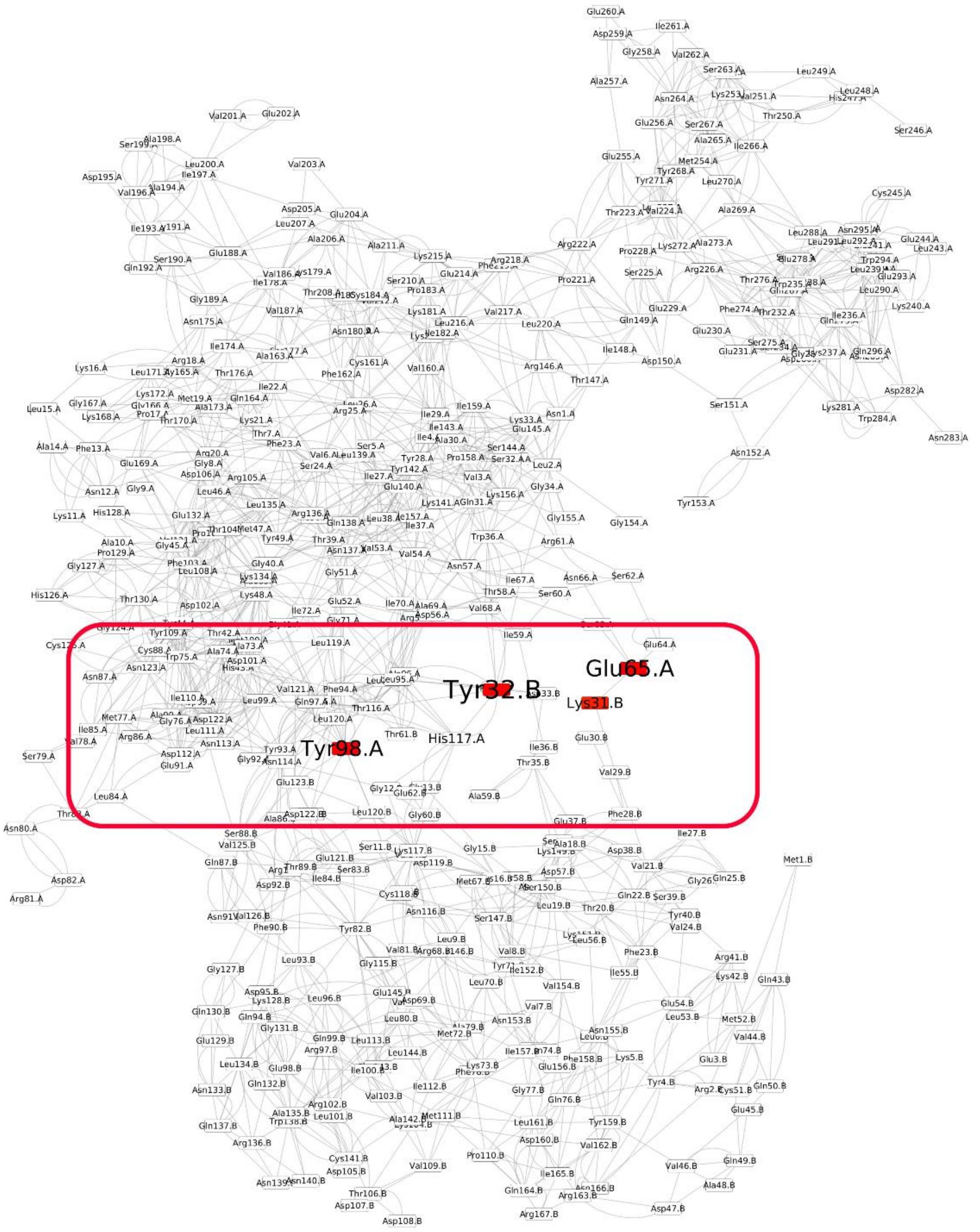

Figure S4. RCA of the interaction between TRPM8 and Rap1A.

The numbering here is slightly modified for TRPM8 by 142 thus His117 is actually His259. Colored in red are the residues considered as central (Z score ≥ 3) in at least one of the five selected docking models. Only central residues at the interface are colored. Chain A corresponds to TRPM8 while B is used for Rap1A.

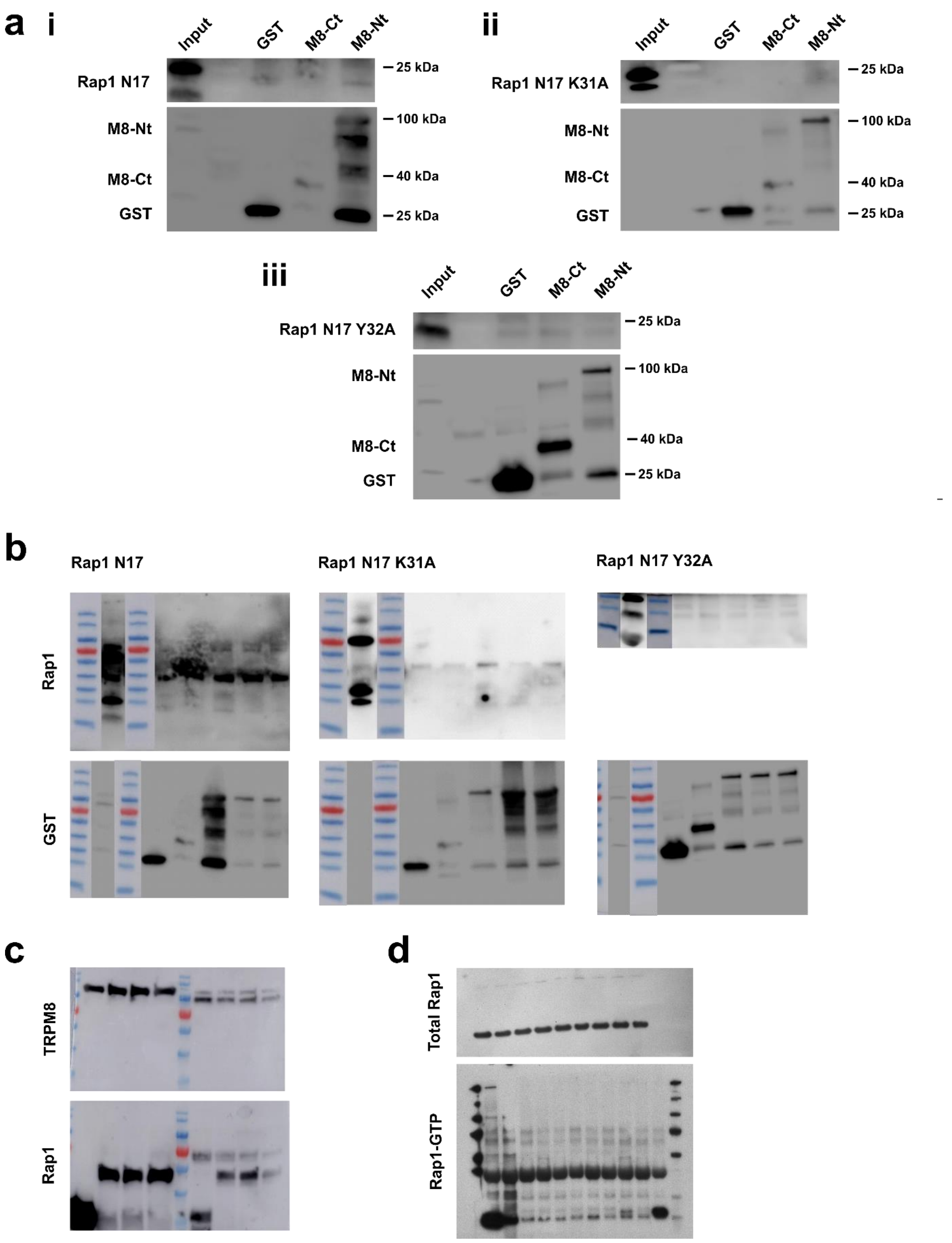

Figure S5. Role of K31 and Y32 in mediating the direct interaction between the inactive (GDP-bound) form of Rap1 and TRPM8 N terminus.

a) GST blots relative to GST pull-down assay described in Fig. 5a (GST-TRPM8 N-terminal tail (M8-Nt), GST-C-terminal tail (M8-Ct), or GST incubated with *in vitro* translated Rap1 N17 (**i**), GFP-Rap1 N17 K31A (**ii**) and GFP-Rap1 N17 Y32A (**iii**). The interaction of Rap1 mutants with GST-fused TRPM8 N- and C-terminal tail was detected using anti-Rap1 antibody and anti-GST antibody was used as control for the loading of GST-fused TRPM8 N- and C-terminal tail. One representative experiment of three (the same shown in fig. 5a) is shown.

b) Original uncropped blots referring to GST pull-down assays reported in Fig. 5ai, 6ai, aii, and aiii; samples order (from left to right): 1) MW marker; 2) input; 3) MW marker; 4) GST; 5) TRPM8 Ct-GST; 6) TRPM8 Nt-GST; 7) TRPM8 E207A Nt-GST; 8) TRPM8 Y240A Nt-GST.

c) Original uncropped blots referring to Co-IP experiment reported in Fig. 5bi; samples order (from left to right): 1) MW marker; 2) GFP (total lysates TL); 3) Rap1 N17-GFP (TL); 4) Rap1 N17 K31A-GFP (TL); 5) Rap1 N17 Y32A-GFP (TL); 6) MW marker; 7) GFP (immuno-precipitated IP); 8) Rap1 N17-GFP (IP); 9) Rap1 N17 K31A-GFP (IP); 10) Rap1 N17 Y32A-GFP (IP).

d) Original uncropped blots referring to the active Rap1 pull-down assays reported in Fig. 5ci and 5ei; samples order (from left to right): 1) GTP Ƴs: 2) GDP; 3) TRPM8+Rap1 N17 Y32; 4) TRPM8+Rap1 N17; 5) TRPM8; 6) Rap1 N17 Y32; 7) Rap1 N17; 8) PC3 CNTRL; 9) Rap1 wt; 10) Rap1 K31A; 11) Rap1 Y32A.

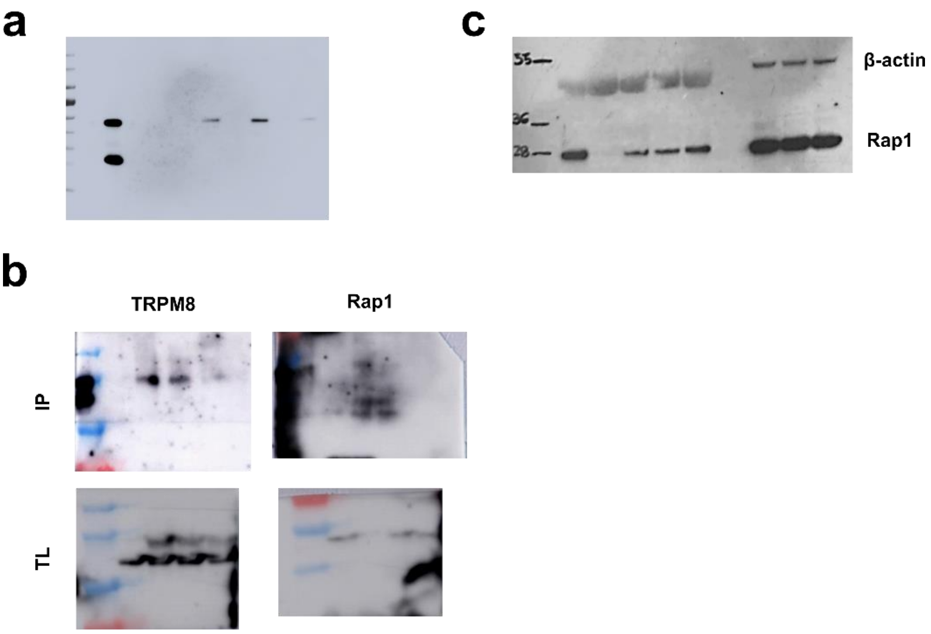

Figure S6. Original uncropped blots referring to Fig. 6 and 7

a) Original uncropped blots referring to GST pull-down assays reported in Fig. 6bi; samples order (from left to right): 1) MW marker; 2) input; 3) GST; 4) TRPM8 Ct-GST; 5) TRPM8 Nt-GST; 6) TRPM8 E207A Y240A Nt-GST.
b) Original uncropped blots referring to Co-IP experiment reported in Fig. 6ci; samples order (from left to right): 1) MW marker; 2) Rap1 N17; 3) TRPM8; 4) TRPM8+Rap1 N17; 5) TRPM8 E207A Y240A+Rap1 N17.
c) Original uncropped blots referring to the active Rap1 pull-down assays reported in Fig. 7di; samples order (from left to right): 1) GTP Ɣs: 2) GDP; 3) PC3 CNTRL (pulled-down PD); 4) TRPM8 (PD); 5) TRPM8 E207A Y240A (PD); 6) empty; 7) PC3 CNTRL (total lysates TL); 8) TRPM8 (TL); 9) TRPM8 E207A Y240A (TL)

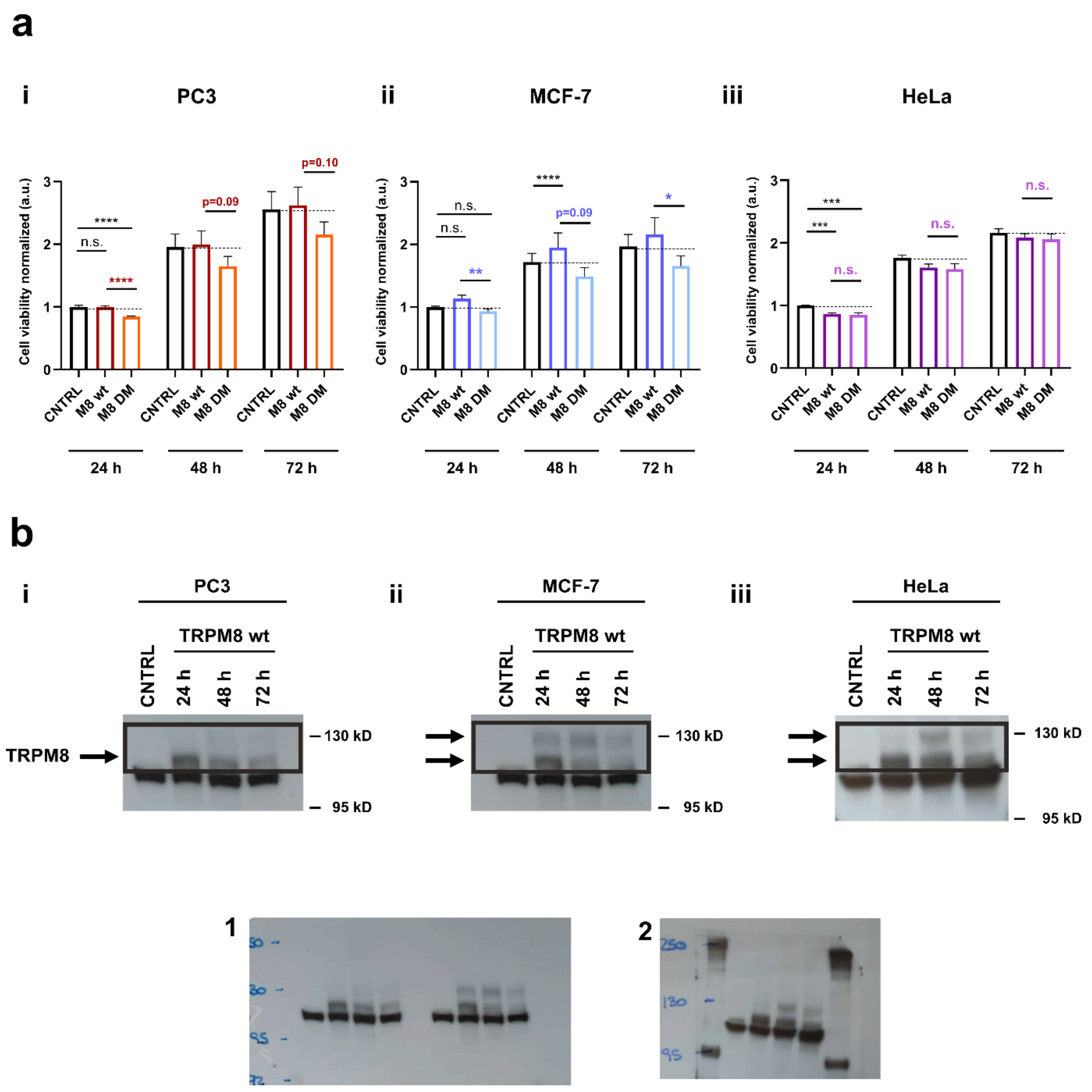

Figure S7. TRPM8-Rap1A interaction in cancer cells viability.

(**a**) Cell viability of PC3 (**i**), MCF-7 (**ii**) and HeLa (**iii**) cells 24, 48, and 72 h after transfection with 0.625 µg of TRPM8 wt or TRPM8 E207A Y240A (M8 DM). Data are normalized on the CNTRL (cells transfected with empty vector) at 24 h and are represented as mean ± SEM. Data refer to a pool of 3 independent experiments (eight replicates for each experiment). Statistical significance: * $p< 0.05$, ** $p< 0.01$, *** < 0.001 **** $P< 0.0001$ (Kruskal-Wallis test with post-hoc Dunn's test).

(**b**) Immunoblot showing TRPM8 overexpression (rabbit anti-TRPM8 Ab109308 1:800) in PC3 (i), MCF-7 (ii) and HeLa (iii) cells 24, 48, and 72 h after transfection with 0.625 µg of TRPM8 wt; The specific bands referring to TRPM8 expression are framed and indicated by the arrows; the original uncropped blots are reported below; samples order in blot 1 (from left to right): 1) PC3 wt; 2) PC3+TRPM8 24 h after transfection; 3) PC3+TRPM8 48 h after transfection; 4) PC3+TRPM8 72 h after transfection; 5) empty; 6) MCF-7 wt; 7) MCF-7+TRPM8 24 h after transfection; 8) MCF-7+TRPM8 48 h after transfection; 9) MCF-7+TRPM8 72 h after transfection; ; samples order in blot 2 (from left to right): 1) HeLa wt; 2) HeLa+TRPM8 24 h after transfection; 3) HeLa+TRPM8 48 h after transfection; 4) HeLa+TRPM8 72 h after transfection.

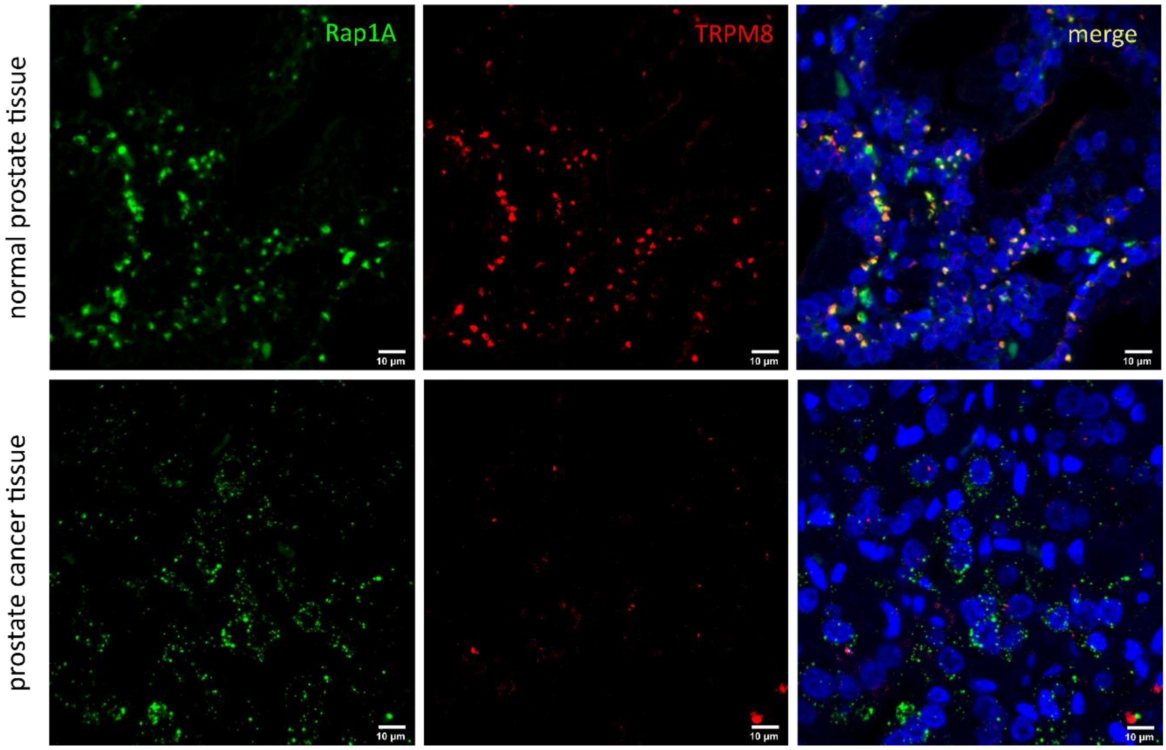

Figure S8. TRPM8-Rap1A interaction in healthy and cancerous prostate tissues.

Representative confocal micrographs of healthy prostate tissue and prostate adenorcarcinoma (Gleason 5) microarrays (Biomax Inc.) stained for Rap1A (green - anti-Rap1A ABIN2854404) and TRPM8 (red - anti-TRPM8 ABIN572229). Nuclei are counterstained with DAPI (blue). Scale bar: 10 µm
